# Effects of dietary methionine and cysteine restriction on plasma biomarkers, serum fibroblast growth factor 21, and adipose tissue gene expression in women with overweight or obesity: a double-blind randomized controlled pilot study

**DOI:** 10.1186/s12967-020-02288-x

**Published:** 2020-03-11

**Authors:** Thomas Olsen, Bente Øvrebø, Nadia Haj-Yasein, Sindre Lee, Karianne Svendsen, Marit Hjorth, Nasser E. Bastani, Frode Norheim, Christian A. Drevon, Helga Refsum, Kathrine J. Vinknes

**Affiliations:** 1 Department of Nutrition, Institute of Basic Medical Sciences, University of Oslo, Postboks 1046, Blindern, 0317 Oslo, Norway; 2 The Lipid Clinic, Department of Endocrinology, Morbid Obesity and Preventive Medicine, Oslo University Hospital, OUS HF Aker Sykehus, Postboks 4959, Nydalen, 0424 Oslo, Norway

**Keywords:** Methionine restriction, Dietary intervention, Plasma biomarkers, Translational research, Adipose tissue, mRNA expression

## Abstract

**Background:**

Dietary restriction of methionine and cysteine is a well-described model that improves metabolic health in rodents. To investigate the translational potential in humans, we evaluated the effects of dietary methionine and cysteine restriction on cardiometabolic risk factors, plasma and urinary amino acid profile, serum fibroblast growth factor 21 (FGF21), and subcutaneous adipose tissue gene expression in women with overweight and obesity in a double-blind randomized controlled pilot study.

**Methods:**

Twenty women with overweight or obesity were allocated to a diet low (Met/Cys_-low,_ n = 7), medium (Met/Cys_-medium,_ n = 7) or high (Met/Cys_-high,_ n = 6) in methionine and cysteine for 7 days. The diets differed only by methionine and cysteine content. Blood and urine were collected at day 0, 1, 3 and 7 and subcutaneous adipose tissue biopsies were taken at day 0 and 7.

**Results:**

Plasma methionine and cystathionine and urinary total cysteine decreased, whereas FGF21 increased in the Met/Cys_-low_ vs. Met/Cys_-high_ group. The Met/Cys_-low_ group had increased mRNA expression of lipogenic genes in adipose tissue including *DGAT1*. When we excluded one participant with high fasting insulin at baseline, the Met/Cys_-low_ group showed increased expression of *ACAC, DGAT1,* and tendencies for increased expression of *FASN* and *SCD1* compared to the Met/Cys_-high_ group. The participants reported satisfactory compliance and that the diets were moderately easy to follow.

**Conclusions:**

Our data suggest that dietary methionine and cysteine restriction may have beneficial effects on circulating biomarkers, including FGF21, and influence subcutaneous adipose tissue gene expression. These results will aid in the design and implementation of future large-scale dietary interventions with methionine and cysteine restriction.

*Trial registration* ClinicalTrials.gov Identifier: NCT03629392, registration date: 14/08/2018 https://clinicaltrials.gov/ct2/show/NCT03629392.

## Background

Overweight and obesity are major risk factors for metabolic disruptions including dyslipidemia and insulin resistance that increase overall risk of morbidity and mortality [1, 2]. Dietary intervention is a common strategy in obesity management; however, whether specific dietary interventions are more efficient than others for weight control is still debated. Dietary restriction of the sulfur amino acids methionine and cysteine has the past decades become increasingly recognized as a research model to promote leanness and longevity in animals [3–6].

Methionine is an essential amino acid that through a sequence of reactions can be converted to cysteine [7]. Cysteine is a precursor of the antioxidant glutathione, and taurine via catabolism by the enzyme cysteine dioxygenase (CDO). In human observational studies, plasma methionine and total cysteine (tCys) are associated with body mass index (BMI), fat mass, insulin resistance and markers of the activity of the lipogenic enzyme stearoyl coenzyme A desaturase (SCD) [8–11]. In rodents, dietary restriction of methionine and cysteine are associated with several beneficial metabolic effects including increased energy expenditure, enhanced insulin sensitivity and decreased adiposity [12–15]. One of the primary biological responses includes effects on lipid metabolism such as increased expression of genes involved in de novo lipogenesis, lipolysis and fatty acid oxidation in liver and white adipose tissue [13, 16, 17]. The available literature from preclinical work support that fibroblast growth factor 21 (FGF21), a member of the endocrine FGF superfamily, is one of the main factors that regulate lipid and glucose metabolism in methionine restricted mice [18–20].

Despite the clear benefits of methionine and cysteine restriction in animal experiments, and epidemiologic data suggesting that cysteine is obesogenic [21], there are few controlled dietary interventions with methionine and cysteine restriction in humans. One dietary intervention with severe restriction of methionine (> 80% relative to controls), but not cysteine, in subjects with metabolic syndrome (n = 26), demonstrated increased fat oxidation compared to the control group, whereas there were no differences in cardiometabolic risk markers and body composition [22]. The lack of effects in the intervention group in this study might be due to cysteine intakes as indicated by reversal of the beneficial effects of methionine restriction by cysteine supplementation [13]. To our knowledge, no studies have focused on effects of combined methionine and cysteine restriction in overweight and obese populations. Furthermore, no studies have evaluated whether moderate reduction (~ 35% relative to controls), may have similar metabolic effects and be a more feasible approach both in research settings as well as in everyday life.

The primary aim of this pilot study was to evaluate the potential effects of a 7-day diet low, moderate or high in methionine and cysteine content on a wide selection of potential outcomes identified in preclinical and observational studies [9, 13, 14, 16, 18] that may aid in the planning and design of a future large-scale study. Outcome measures included plasma lipids, amino acids, glucose, serum FGF21, and other biomarkers related to energy metabolism, including adipose tissue gene expression and plasma SCD activity indices in healthy women with overweight and obesity.

## Materials and methods

### Study design

**Fig. 1:**
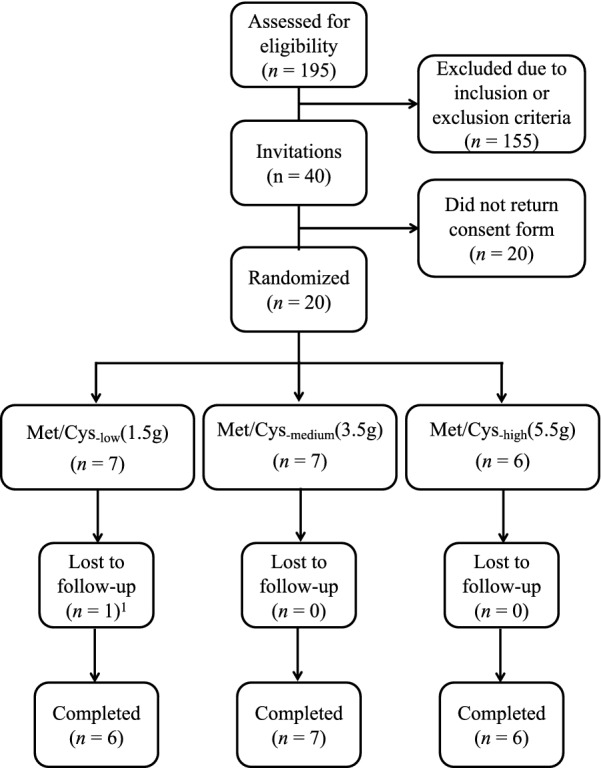
Flow chart of the study participants and progression. The Met/Cys groups had low, medium or high intake of methionine and cysteine. ^1^One participant did not complete the last visit (V4), but all data obtained at V1–V3 were included in relevant analyses

We conducted a 7-day, double-blind, randomized controlled pilot intervention study at the Centre for Clinical Nutrition at Institute of Basic Medical Sciences, University of Oslo, Norway from October 2018 until February 2019. The participants were recruited through the social media channels of the Faculty of Medicine and the Department of Nutrition, University of Oslo. Of the 195 participants that were assessed for eligibility, 20 were randomly assigned, and 19 completed all study visits (Fig. 1). The participants were healthy women with overweight or obesity (BMI 25–35 kg/m^2^) aged 20–40 years. Participants were also included if body fat percentage corresponded to > 33% as measured by dual-energy X-ray absorptiometry [23]. Exclusion criteria were presence of major chronic disease, use of medications, smoking, strenuous physical activity ≥ 3 times/week, veganism, pregnancy, and breastfeeding. Because this was a pilot study where we did not have sufficient information on relevant effects from previous studies, no formal power calculations were performed. The number of participants were based on expected participant recruitment applying the same strategies as in our previous pilot study [24]. Results from this study were reported in accordance with the CONSORT guidelines for pilot studies [25, 26].

The participants were randomly assigned in a 1:1:1 manner to 1 of 3 interventions groups that were severely restricted (Met/Cys_-low_), moderately restricted (Met/Cys_-medium_), or high in methionine and cysteine (Met/Cys_-high_) content. Randomization and assignment were performed by researchers unrelated to the study using the “blockrand” package in R (R for statistical computing, Vienna). A block size of 12 was used to ensure similar numbers of participants per group. Thus, the study personnel were blinded to group allocation. Details of the composition of the diets are described below. Blood sampling were performed at baseline (visit 1), and after 1 (visit 2), 3 (visit 3) and 7 (visit 4) days. Clinical and anthropometric measurements and fat biopsies were performed at visit 1 and visit 4. Before the baseline visit, all participants performed a 1-week run in period with instructions to avoid intake of fatty fish, cod liver oil, n-3 fatty acid supplements and other supplements, to moderate their alcohol consumption (maximum 2 units 3 times per week), and to keep their physical activity level normal. The day before study start, participants were recommended to avoid strenuous physical activity, to abstain from alcohol and fast 10 h before blood sampling.

All participants gave written informed consent. The study was conducted according to the guidelines in the Declaration of Helsinki, and the Regional Ethics Committee for Medical Research in South East Norway approved the study (reference number: 2015/634). The study was registered at US National Library of Medicine Clinical Trials registry (ClinicalTrials.gov Identifier: NCT03629392, registration date: 14/08/2018).

### Dietary interventions

The diet was based on the intervention in a previous pilot study [24] with some modifications. The diet was adjusted to the Nordic Nutrient Recommendations for females aged 18–60 years with an average energy requirement corresponding to about 2200 kcal/d [27]. Because the main aim of the study was to compare diets contrasting in sulfur amino acid content, the total number of calories were the same for each participant. A detailed overview of the nutrient composition of the diet is presented in Additional file 1. Meals were developed to fit a Norwegian cuisine and available ingredients. A typical menu is displayed in Additional file 2.

Sulfur amino acids are found in most foods but are especially high in animal products. Thus, the diets were vegan-based and consisted of plant foods. In order to achieve recommended protein and micronutrient intake, all participants were given powdered drink as a supplement to the foods. The supplement contained all essential and non-essential amino acids carbohydrates, vitamins, minerals and trace elements, excluding sulfur amino acids (XMET XCYS Maxamaid^®^, Nutricia Norway AS, Oslo, Norway). Thus, the design of the intervention was double-blind. To improve the flavor of the drink mixes, the participants added a flavoring powder (Flavour Modjul^®^ from Nutricia) with either a blackcurrant or orange taste.

We designed three diets with different sulfur amino acid content by regulating the quantity of methionine and cysteine powder added to the diet. This resulted in a diet with a low (Met/Cys_-low_), medium (Met/Cys_-medium_) or high (Met/Cys_-high_) sulfur amino acid content. The Met/Cys_-high_ group was considered the control group and were based on intake estimates on high protein diets [28]. The Met/Cys_-low_ and Met/Cys_-medium_ groups were selected based on an overall aim to achieve severe and moderate restriction as compared with the Met/Cys_-high_ group. All diets fulfilled the minimum WHO recommendation of daily intake of sulfur amino acids of 15 mg/kg [29], and corresponded to a content that was on average 72% and 35% relative to the Met/Cys_-high_ group. No powder was added to the Met/Cys_-low_ diet; thus, the mean content of sulfur amino acids in the diet was 1.6 g/day (0.8 g/d of methionine and 0.8 g/d of cysteine). An overview can be found in Additional file 3. In the Met/Cys_-medium_ diet, we added a total of 2.04 g of powder (0.68 g of methionine and 1.36 g cysteine) resulting in a mean daily intake of 3.64 g/d (1.48 g/d of methionine and 2.16 g/d of cysteine). In the Met/Cys_-high_ diet we added 4 g of powder (1.32 g of methionine and 2.68 g of cysteine), with a total mean intake of 5.6 g/d of (2.12 g/day of methionine and 3.48 g/d of cysteine).

All foods and supplements, including a menu with recipes for each meal and information on daily intake, were supplied to the participants and were delivered to their home address by a delivery service.

### Anthropometric parameters

A bioelectrical impedance weight (Seca mBCA515, Hamburg, Germany) was used to measure body weight and calculate BMI.

### Blood and urine sampling and biochemical assays

Venous blood samples were collected from each participant at days 0, 1, 3 and 7 after an overnight fast. Description of the blood sampling, and amino acids assays has been reported in details previously [24]. Blood was collected into 2 EDTA-lined vacuum tubes for measurements of total amino acids, total free (unbound), and free oxidized and reduced homocysteine, cysteine and glutathione, and S-adenosylmethionine and S-adenosylhomocysteine. To trap the thiols, a tube contained N-ethylmalemide 150 mmol/L at 10% of the volume of the tube [30]. Immediately after withdrawal, the blood was centrifuged for 5 min at 4 °C, followed by precipitation with 5-sulfosalicyclic acid 10%, with the resultant supernatant aliquoted and stored at − 80 °C until analysis.

Blood samples for measurement of total cholesterol, low-density lipoprotein cholesterol (LDL-C), high-density lipoprotein cholesterol (HDL-C) and triglycerides, apolipoprotein A1 (apoA1), apolipoprotein B (apoB), glucose, and insulin were measured at Department of Medical Biochemistry (Oslo University Hospital Rikshospitalet, Oslo, Norway) by colorimetric and/or enzymatic methods (Cobas c702 analyzer, Roche Diagnostics International Ltd, Rotkreuz, Switzerland). Serum FGF21 concentrations were measured by the R&D Systems Human FGF-21 Quantikine ELISA with intra-assay CV: 2.9–3.9% and inter-assay CV: 5.2–10.9%.

### Amino acid assays

Total (plasma and urine), free reduced/oxidized (plasma) and unbound (plasma) methionine, homocysteine, cysteine, cystathionine, taurine and glutathione as well as S-adenosylmethionine and S-adenosylhomocysteine were measured by liquid chromatography–tandem mass spectrometry (LC–MS/MS) as described in detail in a previous publication [24]. Coefficients of variation for total plasma and urinary sulfur amino acids were 3.4–6.7%. For plasma SAM and SAH, the coefficients of variation were 2–3%, and 4–6% for the total unbound fraction representing the combined free reduced and disulfide concentrations. The bound fraction was calculated by subtracting the unbound concentration from the total plasma concentration.

### Fatty acid assays

Plasma samples, thawed in fridge overnight, were vortexed, centrifuged and pipetted into vials. Internal standard (triheptadecanoin) was added and samples were methylated with 3 N HCl in methanol. FAMEs were extracted with hexane, then samples were neutralized with 3 N KOH in water. After mixing and centrifuging the hexane phase was injected into the GC-FID. Analysis was performed on a 7890A GC with a split/splitless injector, a 7683B automatic liquid sampler, and flame ionization detection (Agilent Technologies, Palo Alto, CA). Separations were performed on a SP-2380 (30 m × 0.25 mm i.d. × 0.25 µm film thickness) column from Supelco. As a surrogate marker of liver SCD1 activity, we examined the plasma SCD-16 index estimated by the product/precursor-ratio of fatty acids in plasma (C16:1n-7/C16:0).

### Diet feasibility and evaluation

The participants’ subjective rating and evaluation of the diets were collected with questionnaires that included visual analog scales providing a range of scores from 0 to 100, with 0 = “no pain/symptoms/great satisfaction” and 100 = “maximum pain/symptom/dissatisfaction”.

### Subcutaneous fat tissue biopsies

Subcutaneous adipose tissue was obtained from the periumbilical region by a suction-based biopsy procedure. The skin was sterilized and a half-circular area of skin and subcutis were anaesthetized by injecting 5 mL of a local anesthetic (Xylocain 10 mg/mL AstraZeneca, Södertälje, Sweden). A needle (Sterican 2.10 × 80, Ref. No. 04665473, B Braun, Melsungen, Germany) connected to a vacuum syringe was inserted into subcutaneous adipose tissue. Subcutaneous adipose tissue biopsies were then dissected on an ice-cold aluminum plate to remove blood and other materials before aliquots were subsequently snap-frozen in 1.5 mL cryotubes and stored at − 80  °C.

### Isolation of RNA, complementary DNA synthesis and quantitative real-time PCR

RNA from frozen fat tissue was extracted using TRIzol (thermoFisher) and RNeasy lipid Tissue mini kit (Qiagen) according to the manufacturer instructions. The quantity and quality of the RNA were monitored using a Nanodrop ND-1000 Spectrophotometer (Thermo Fisher Scientific, Waltham, MA, US). A RIN value of > 7.0 was considered acceptable to proceed wth qPCR analysis.

Isolated RNA (250 ng) was reversely transcribed to complementary DNA using the High-Capacity cDNA Reverse Transcription kit (Applied Biosystems). Quantitative real-time PCR (qPCR) was performed with either 2.5 µL diluted cDNA (12.5 ng RNA) mixed in a 10 µL Kapa SYBR FAST qPCR Master Mix Universal (KapaBiosystems, Wilmington, MA, US), or 9 µL diluted cDNA (25 ng RNA) and 1 µL predeveloped TaqMan Gene Expression Assays were mixed in 10 µL TaqMan Gene Expression Master Mix (Thermo Fisher Scientific, Waltham, MA, US), on a Bio-Rad CFX96 Touch™ Real-Time PCR Detection System (Bio-Rad Laboratories, Hercules, CA, US).

Assay primers for Kapa SYBR FAST qPCR reactions were designed with Primer-BLAST software (NCBI, Bethesda, MD, US). Primer sequences are listed in Additional file 4. Predeveloped primers and probe sets (TaqMan assays) were used to analyse mRNA levels of the following target genes (official gene symbol in parenthesis): Leptin (*LEP*), Hs00174877_m1; PR/SET domain 16 (*PRDM16*), HS00922674_m1; and ribosomal protein lateral stalk subunit P0 (RPLP0), HS9999902_m1. Relative target mRNA expression was normalized to one out of the two housekeeping genes, TBP and RPLP0, and quantified using the ∆∆^Ct^ method.

### Outcomes

The primary outcomes were changes in plasma concentrations of sulfur amino acids and mRNA expressions relevant to sulfur amino acid and lipid metabolism in adipose tissue. In the original protocol, we specified protein expression as primary outcomes, but due to methodological challenges we decided to initially proceed with mRNA analyses. We included mRNAs shown to be affected by methionine and cysteine restriction in preclinical studies [31]. In addition, we included mRNAs involved in the regulation of cysteine metabolism to evaluate whether compensatory mechanisms are activated when dietary intake of methionine and cysteine is restricted as has been reported in animal models [31–33]. Finally, we assessed FGF21, the SCD-16 index as well as the self-reported compliance and feasibility of the diet.

### Statistical analysis

Null-hypothesis testing is actively discouraged in pilot trials, however, because we have screened a wide range of potential outcomes with relevance for a future large-scale study, we have reported p-values and confidence intervals (CI), but stress that these estimates should be interpreted with care as per the CONSORT statement for reporting of clinical trials and pilot trials [25, 26]. Findings with p > 0.05 but with CIs indicative of the direction of the effect, and in line with reports from preclinical studies, were considered meaningful for the design of a full-scale study. Based on the CONSORT statement for reporting of clinical trials [34] as well as recommendations from Altman and others [35–38], we have not performed significance tests for baseline covariates.

The plasma amino acid and serum FGF21 concentrations were repeatedly measured (day 0, 1, 3 and 7), and we used linear mixed regression models to adjust for within-subject variability. The models included the respective amino acids in plasma or serum FGF21 as the dependent variable, and group, time and their interaction term (group × time) as fixed, independent variables. Subject ID was added to the model as a random effect. A quadratic term of time (time^2^) and its interaction with group was added to the outcome models including amino acids (group × time^2^). In addition, due to the small sample size we expected baseline differences and the models including amino acids and FGF21 were adjusted accordingly. An unstructured covariance structure was assumed based on Akaike’s information criterion. The model residuals were normally distributed, and we used the raw data for analysis. Finally, because we compared the Met/Cys_-low_ and Met/Cys_-medium_ with Met/Cys_-high_ independently, separate models were constructed. The main outcome analyses were plotted using the mean linear predictions and individual predicted points from the linear mixed models and the *p* value for interaction.

Baseline demographic and routine laboratory measurements are presented as medians (ranges). The effect of the dietary intervention on routine laboratory variables and the SCD-16 index were assessed with analysis of covariance (ANCOVA) adjusted for baseline concentrations of the covariates. The model residuals were not normally distributed as assessed visually by histograms, and thus we log-transformed the covariates prior to analysis, which resulted in normally distributed model residuals. The results are presented in tables as back-transformed estimated marginal mean difference from the Met/Cys_-high_ group with corresponding CI and p-values. Because the estimates are back-transformed, they are given in  %. Comparison of changes in mRNA expression were performed on log_2_-transformed 2^−ΔCT^ values and were assessed with ANCOVA adjusted for differences in baseline expression. The Met/Cys_-low_ and Met/Cys_-medium_ groups were compared against the Met/Cys_-high_ group which served as the reference group. Estimates are presented as log_2_-fold change with individual points. We identified one outlier with very high homeostatic model of insulin resistance (HOMA-IR) values at baseline (> 6.0). Because insulin can have profound effects on the subcutaneous adipose tissue, we presented analyses with and without this participant. We performed additional analyses adjusted for apparent imbalances due to unfortunate randomization for age, BMI and triglycerides. Because the inclusion of these covariates did not alter effect estimates materially we did not include them in the final models.

We calculated Spearman`s correlation coefficients and the CI between the sulfur amino acids and subcutaneous adipose tissue mRNA expression.

## Results

### Study participants

Of 40 women invited to participate, 20 were randomized to the intervention groups and 19 completed the trial (Fig. 1). The participants (23–40 years of age), had BMI ranging from 24.7 to 34.7 kg/m^2^ (Table 1).

**Table 1:** Baseline characteristics of the study population

Characteristics	Met/Cys-low	Met/Cys-medium	Met/Cys-high
	(n = 7)	(n = 7)	(n = 6)
Age, y	29.5 (24.9, 40.9)	30.9 (23, 39)	34.5 (23.7, 39.7)
Body weight (kg)	83.2 (76, 108)	81.8 (73, 97)	78.3 (68.4, 83.3)
BMI (kg/m^2^)	29.7 (25.5, 34.7)	29.4 (24.7, 32.9)	27.3 (24.4, 33.8)
Triglycerides (mmol/L)	0.9 (0.6, 1.1)	0.8 (0.5, 2)	1.45 (0.7, 2.1)
Total cholesterol (mmol/L)	3.9 (3.7, 4.6)	4.4 (4, 6)	4.55 (3.6, 6.3)
LDL cholesterol (mmol/L)	2.4 (1.7, 3.2)	2.8 (2.6, 4.5)	2.65 (2, 4.9)
HDL cholesterol (mmol/L)	1.4 (0.9, 2.1)	1.4 (0.8, 1.9)	1.5 (1.1, 1.9)
Apolipoprotein B (g/L)	0.8 (0.6, 1)	0.8 (0.7, 1.2)	0.8 (0.6, 1.4)
Apolipoprotein A1 (g/L)	1.4 (1, 2.1)	1.4 (1, 1.7)	1.5 (1.2, 1.9)
Fasting glucose (mmol/L)	5 (4.6, 5.8)	5 (4.5, 5.3)	5.15 (4.1, 5.6)
Fasting insulin (pmol/L)	54 (30, 162)	44 (32, 62)	54.5 (21, 60)
Fasting C-peptide (pmol/L)	563 (385, 1220)	614 (481, 740)	614 (458, 832)
HOMA-IR^a^	2.08 (1.2, 6)	1.63 (1.07, 2.25)	1.94 (0.78, 2.31)
FGF21 (pg/mL)	95.9 (37.8, 312)	98.0 (67.3, 232)	75.0 (5.0, 291)
*Amino acids (μmol/L)*			
Methionine	19.9 (18.2, 25.5)	21.4 (19.5, 24.4)	22.3 (19.2, 26.4)
SAM	101 (69.9, 114)	94.9 (84.5, 109)	103 (89, 129)
SAH	24.7 (17.3, 29.7)	24.7 (20.4, 28.1)	25.8 (22.8, 35.3)
Total homocysteine	8.86 (6.56, 9.68)	9.99 (5.03, 14.8)	7.04 (4.66, 11.2)
Free homocysteine	1.23 (0.814, 2.09)	1.84 (0.942, 2.9)	1.26 (0.79, 2.32)
Free reduced homocysteine	0.17 (0.14, 0.48)	0.38 (0.17, 0.42)	0.18 (0.17, 0.26)
Homocystine	0.02 (0.02, 0.03)	0.03 (0.01, 0.06)	0.02 (0.01, 0.03)
Protein-bound homocysteine	7.47 (5.58, 8.22)	8.17 (4.08, 11.9)	5.85 (3.87, 8.87)
Cystathionine	1.6 (0.80, 2.91)	1.32 (0.69, 2.32)	1 (0.65, 3.48)
Total cysteine	310 (288, 322)	286 (248, 331)	291 (261, 324)
Free cysteine	152 (119, 178)	161 (146, 176)	169 (140, 179)
Free reduced cysteine	18 (11.4, 26.8)	20.2 (14.4, 26.9)	19.2 (11.9, 24.5)
Cystine	44.8 (41, 49.6)	41.9 (41.3, 47.8)	44.5 (42.5, 47.2)
Protein-bound cysteine	149 (134, 189)	123 (99.5, 170)	139 (82, 150)
Total GSH	7.69 (5.66, 7.99)	7.97 (4.46, 10.9)	8.25 (5.43, 13.5)
Free GSH	3.59 (2.39, 6.13)	4.29 (2.49, 6.89)	6.66 (4.1, 10.6)
GSH	4.88 (3.37, 5.85)	5.26 (5.05, 6.21)	6.5 (4.66, 6.91)
GSSG	0.06 (0.03, 0.09)	0.05 (0.02, 0.10)	0.09 (0.05, 0.15)
Protein-bound GSH	3.14 (1.16, 4.58)	2.89 (1.98, 4.69)	2.1 (0.27, 6.01)
Taurine	95.4 (55.3, 126)	98.7 (66.1, 112)	101 (51.1, 136)

Values are given as median (range)

FGF21, fibroblast growth factor 21; Met/Cys, methionine and cysteine; SAM, S-adenosylmethionine; SAH, S-adenosylhomocysteine; GSH, glutathione

^a^HOMA-IR was calculated by (insulin/6) × (glucose × 18)/405

### Changes in body weight and plasma biomarkers

Median (range) body weight in kg were 83.2 (76, 108), 81.8 (73, 97) and 78.3 (68.4, 83.3) at baseline in the Met/Cys_-low_, Met/Cys_-medium_ and Met/Cys_-high_ groups, respectively. At the final visit, the median (range) body weight in kg were 83.2 (75.0, 106), 81.6 (72.0, 97.0) and 77.5 (68.2, 82.0). As expected due to the short duration of the study, there were no conclusive differences in weight change between groups (Table 2). We observed trends for an increase in apoA1 and a decrease in the ratio of apoB to apoA1 and insulin in the Met/Cys_-low_ compared with the Met/Cys_-high_ group (Table 2), but as indicated by the CIs these results were inconclusive.

**Table 2:** Change in markers from baseline to final study visit

Plasma marker	β for Met/Cys_-low_ vs. Met/Cys_-high_	*p*	β for Met/Cys_-medium_ vs. Met/Cys_-high_	*p*
ApoA1	6.16 (− 3.33, 16.6)	0.19	− 4.05 (− 12.4, 5.04)	0.35
ApoB	− 7.21 (− 22.4, 11)	0.39	− 3.08 (− 18.3, 15)	0.70
ApoB/A1	− 13.3 (− 28.4, 4.88)	0.13	− 0.9 (− 17.7, 19.3)	0.92
Total cholesterol	− 1.7 (− 13.2, 11.4)	0.78	− 4.62 (− 14.8, 6.8)	0.39
LDL	− 5.25 (− 21.2, 14)	0.54	− 6.69 (− 21.6, 11.1)	0.41
HDL	2.64 (− 5.26, 11.2)	0.50	− 0.68 (− 8.07, 7.3)	0.85
Triglycerides	8.51 (− 24, 55)	0.63	− 4.54 (− 31.7, 33.4)	0.77
C-peptide	− 1.2 (− 19.5, 21.2)	0.90	− 4.19 (− 21.2, 16.5)	0.65
Insulin	− 19.4 (− 45.6, 19.6)	0.26	− 10.8 (− 38.1, 28.6)	0.52
Glucose	0.48 (− 5.3, 6.61)	0.87	− 0.73 (− 6.26, 5.13)	0.79
SCD16^a^	− 12 (− 27.8, 7.37)	0.19	− 15.7 (− 30.9, 2.84)	0.087
SCD18^a^	7.16 (− 4.46, 20.2)	0.22	5.98 (− 5.02, 18.3)	0.28
HOMA-IR^b^	− 21.3 (− 47.8, 18.7)	0.23	− 10.3 (− 38.7, 31.2)	0.55
Body weight	− 0.29 (− 1.63, 1.08)	0.66	− 0.48 (1.70–0.75)	0.42

Estimated marginal mean change in markers from baseline to the final study visit. Because all raw data values were log-transformed prior to analysis, the βs are given in units of per cent with corresponding confidence intervals

*Met/Cys* methionine and cysteine, *apoA1* apolipoprotein A1, *apoB* apolipoprotein B, *LDL* low-density lipoprotein, *HDL* high-density lipoprotein, *SCD* stearoyl-CoA desaturase

^a^SCD16, C16:1n-7/C16:0; SCD18, C18:1n-9/C18:0

^b^HOMA-IR was calculated by (insulin/6) × (glucose × 18)/405

### Plasma and urine sulfur amino acids

Spearman’s rank correlation coefficients among the plasma sulfur amino acids and related intermediates are shown in Additional file 5.

**Fig. 2:**
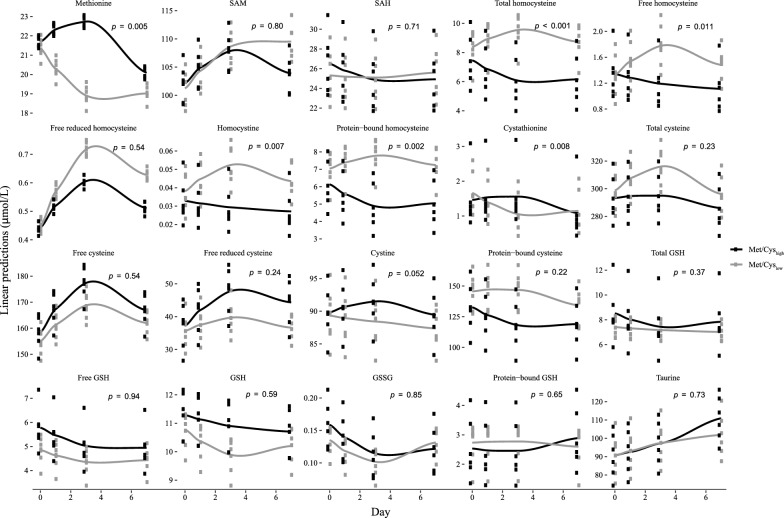
Estimated means linear predictions/response in plasma sulfur amino acids to the diets. Values are derived from a linear mixed model regression. The p-values denote the p for interaction between group and time and indicate the difference in response over time between the Met/Cys_-low_ and Met/Cys_-high_ groups. *Met/Cys* methionine and cysteine, *SAM* S-adenosylmethionine, *SAH* S-adenosylhomocysteine, *GSH* glutathione, *GSSG* oxidized glutathione

To investigate whether the diets induced a response in sulfur amino acids and related compounds, we measured the plasma concentration of the sulfur amino acids and intermediates as well as fractions of total cysteine, homocysteine and glutathione. We observed group × time interactions between the Met/Cys_-low_ and the Met/Cys_-high_ groups for methionine, cystathionine, and total homocysteine and its fractions including bound and free unbound homocysteine, and homocystine (Fig. 2). There was an overall decrease in methionine from baseline to day 7 in the Met/Cys_-low_ group compared with the Met/Cys_-high_ group. For cystathionine the response diverged from baseline to day 7 in the Met/Cys_-low_ group compared with the Met/Cys_-high_ group, but the groups approached near similar concentrations after 7 days. For total homocysteine and its fractions there were overall increases in the response in the Met/Cys_-low_ group compared with the Met/Cys_-high_ group. Of the total cysteine fractions, there was a slight decrease in free oxidized cystine in the Met/Cys_-low_ compared to the Met/Cys_-high_ group.

We calculated Spearman correlation coefficients between plasma methionine, plasma total cysteine, body weight and BMI. There were no correlations between plasma methionine and body weight or BMI at baseline (BMI: *r *= − 0.09, 95% CI − 0.05, 0.37; body weight: − 0.13, 95% CI − 0.54, 0.33) or day 7 (BMI: *r *= 0.31, 95% CI − 0.26, 0.67; body weight: 0.13, 95% CI − 0.34, 0.55). Plasma total cysteine tended to correlate with BMI at baseline (*r *= 0.43, 95% CI − 0.02, 0.73), but not body weight at baseline (*r *= 0.26, 95% CI − 0.21, 0.63); or BMI (*r *= 0.32, 95% CI − 0.16, − 0.68) and body weight at day 7 (*r *= 0.18, 95% CI − 0.30, 0.68).

When comparing the Met/Cys_-medium_ group with the Met/Cys_-high_ group, there were group × time interactions for methionine and cystathionine, with similar responses as in the Met/Cys_-low_ group (Additional file 6).

Urinary concentrations of tCys were reduced in the Met/Cys_-low_ and Met/Cys_-medium_ groups compared to the Met/Cys_-high_ group (Additional files 7 and 8). Moreover, cystathionine concentrations were reduced in the Met/Cys_-low_ compared to the Met/Cys_-high_ group.

### Serum fibroblast growth factor 21

**Fig. 3:**
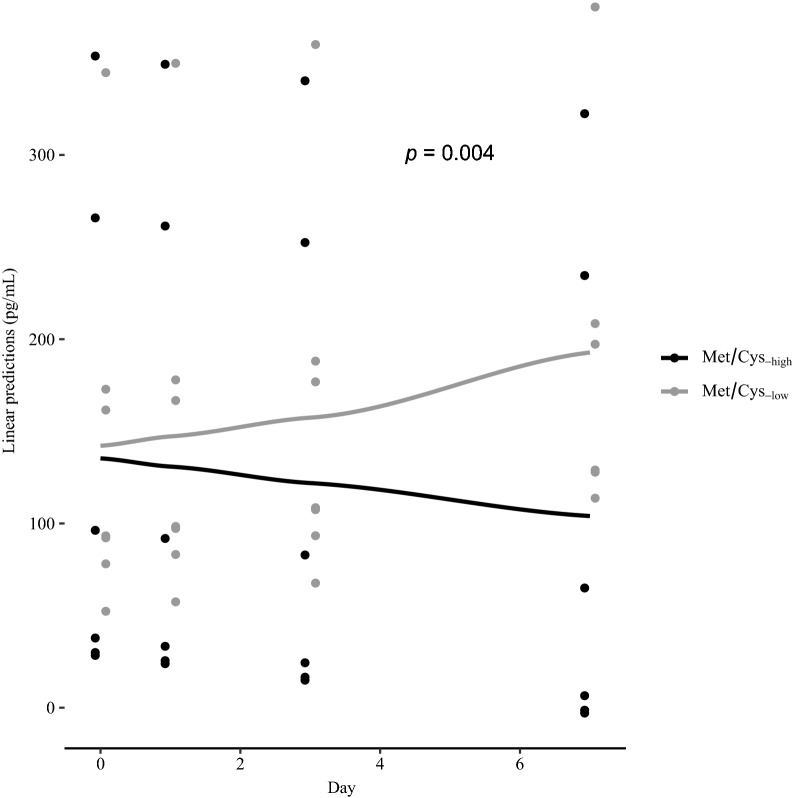
Estimated means linear predictions/response in serum Fibroblast growth Factor 21 to the diets. Values are derived from a linear mixed model regression. The p-values denote the p for interaction between group and time and indicate the difference in response over time between the Met/Cys_-low_ and Met/Cys_-high_ groups. *FGF21* fibroblast growth factor 21, *Met/Cys* methionine and cysteine

The unadjusted median serum FGF21 concentrations increased by 47% in the Met/Cys_-low_ group from baseline to day 7 [median (range) from 96 pg/mL (38, 312) to 141 pg/mL (104, 432)], whereas the concentrations decreased in the Met/Cys_-medium_ and Met/Cys_-high_ groups by 30% [from 98 (67, 232) to 69 (40, 426)] and 64% [from 75 (5, 291) to 27 (10, 332)], respectively. The linear mixed regression analyses revealed a significant group × time interaction for the FGF21 response in the Met/Cys_-low_ group compared with the Met/Cys_-high_ group (Fig. 3), but not for the Met/Cys_-medium_ group versus the Met/Cys_-high_ group (Additional file 9).

### mRNA expression in subcutaneous adipose tissue

**Fig. 4:**
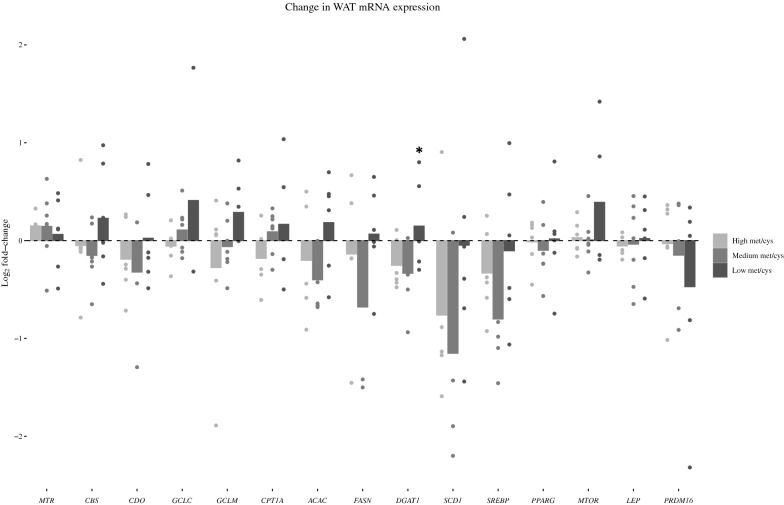
The means log_2_-fold change in mRNA expression of genes in adipose tissue. P-values were derived by analysis of covariance where the Met/Cys_-low_ and Met/Cys_-medium_ groups were compared against the Met/Cys_-high_ group. *p < 0.05 against Met/Cys_-high_. *MTR* methionine synthase reductase, *CBS* cystathionine-β-synthase, *CDO* cysteine dioxygenase, *GCLC* glutamate-cysteine ligase catalytic subunit, *GCLM* glutamate-cysteine ligase regulatory subunit, *CPT1A* carnitine palmitoyl-transferase 1a, *ACACA* acetyl-CoA carboxylase, *FASN* fatty acid synthase, *DGAT1* diacylglycerol *O*-acyltransferase 1, *SCD1* stearoyl CoA-desaturase 1, *SREBP* sterol regulatory element binding protein, *PPARG* peroxisome-proliferator activated receptor γ, *MTOR* mammalian target of rapamycin, *LEP* leptin, *PRDM16* PR domain containing 16

We compared changes in subcutaneous adipose tissue mRNA expression between baseline and day 7, for 16 candidate genes, between the intervention groups (Met/Cys_-low_ and Met/Cys_-medium_) and the control Met/Cys_-high_ group (Fig. 4). Results for each individual can be found in the additional files (Additional files 10, 11, 12). For transcripts related to lipid metabolism, there was an upregulation of diacylglycerol O-Acyltransferase 1 (*DGAT1*), which is involved in synthesis of triacylglycerol, in the Met/Cys_-low_ group, with a 0.43 log_2_-fold change vs. Met/Cys_-high_ after 7 days (95% CI 0.00–0.86). Inconclusive results were observed for transcript levels of acetyl-CoA carboxylase [*ACAC* (0.41 log_2_-fold change vs. Met/Cys_-high_, 95% CI − 0.12–0.94)], *SCD1* (0.87, − 0.29–2.04), glutamate-cysteine ligase catalytic subunit [*GCLC* (0.46, − 0.0–1.01)], glutamate-cysteine ligase modifier subunit [*GCLM* (0.37, − 0.17–0.91)], carnitine palmitoyltransferase I [*CPT1A* (0.33: − 0.15–0.82)], and mammalian target of rapamycin [*MTOR* (0.37, − 0.13–0.88)].

Exclusion of one participant with high baseline HOMA-IR influenced results for transcripts related to lipogenesis including *ACAC* (0.63 log_2_-fold change vs. Met/Cys_-high_, 95% CI 0.19–1.07), and *DGAT1* (0.56, 0.14 –0.99). Similar but inconclusive results were observed for fatty acid synthase [*FASN* (0.58, − 0.11–1.28)], *SCD1* (1.13, − 0.02–2.29), and mammalian target of rapamycin [*MTOR* (0.49, − 0.01–0.99)]. In the Met/Cys_-medium_ group, the response in mRNA expression generally followed the same pattern as the Met/Cys_-high_ group.

### Plasma SCD activity index

As a proxy marker of hepatic SCD1 activity, we examined the plasma SCD-16 index (the ratio between 16:1n-7 and 16:0). The estimated mean changes for the plasma SCD-16 index were compatible with reductions in both the Met/Cys_-low_ (− 12.0%, 95% CI − 27.8%–7.4%) and Met/Cys_-medium_ (− 15.7%, − 30.9–2.84) groups compared to the Met/Cys_-high_ group, but results were inconclusive as indicated by the wide CIs. When we excluded the subject with high HOMA-IR at baseline, results tended towards a greater reduction in the Met/Cys_-low_ compared to the Met/Cys_-high_ group was observed (− 15.4%, − 30.4–3.0%).

### Correlations between plasma sulfur amino acids, fatty acids and subcutaneous adipose tissue mRNA expression

**Fig. 5:**
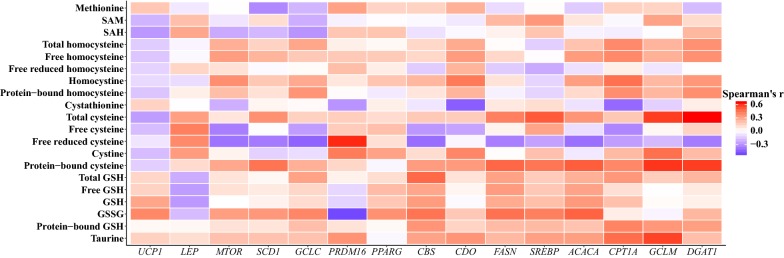
Correlations between changes in plasma sulfur amino acids and mRNA expression of the total population. *SAM* S-adenosylmethionine, *SAH* S-adenosylhomocysteine, *GSH* glutathione, *GSSG* oxidized glutathione, *MTR* methionine synthase reductase, *CBS* cystathionine-β-synthase, *CDO* cysteine dioxygenase, *GCLC* glutamate-cysteine ligase catalytic subunit, *GCLM* glutamate-cysteine ligase regulatory subunit, *CPT1A* carnitine palmitoyl-transferase 1a, *ACACA* acetyl-CoA carboxylase, *FASN* fatty acid synthase, *DGAT1* diacylglycerol *O*-acyltransferase 1, *SCD1* stearoyl CoA-desaturase 1, *SREBP* sterol regulatory element binding protein, *PPARG* peroxisome-proliferator activated receptor γ, *MTOR* mammalian target of rapamycin, *LEP* leptin, *PRDM16* PR domain containing 16

We examined whether changes in plasma sulfur amino acids were correlated with the changes in adipose tissue gene expression (Fig. 5 and Additional file 13). Among the strongest correlations were those for tCys and protein-bound cysteine with *DGAT1* (*r *= 0.64, 95% CI 0.25–0.85; *r *= 0.57, 0.16–0.82, respectively), between tCys and *SREBP* (*r *= 0.51, 0.073–0.78), and between free reduced cysteine and *PRDM16* (*r *=0.61, 0.21–0.83). Other prominent correlations were for the glutathione synthesizing gene, *GCLM,* with tCys (*r *= 0.57, 0.16–0.82), protein-bound cysteine (*r *= 0.59, 0.18–0.82), and taurine (*r *= 0.56, 0.15–0.81).

### Diet feasibility and evaluation

Self-reported compliance with the diets was ~ 95% among the participants, and there were no differences in self-reported compliance between the groups (data not shown). Satisfaction with the diets was moderate (51 points on a 100-point scale), and the participants reported that the diets were moderately easy to follow (39 points). Side effects were light to moderate for gastrointestinal and other symptoms including headache, fatigue, dizziness, and sleep disturbances. There were no differences in side effects between groups (data not shown). No severe harmful effects were reported.

## Discussion

This double-blind randomized controlled pilot study describes for the first time the effects of dietary restriction of both methionine and cysteine content, with either severe or moderate restriction on plasma biomarkers and subcutaneous adipose tissue mRNA expression related to energy metabolism, in healthy overweight and obese women. Participants in the Met/Cys_-low_ group tended towards improvements in plasma apoB/A1 after 7 days of intervention, but the results were inconclusive. In addition, FGF21 increased in the Met/Cys_-low_ group compared to the Met/Cys_-high_ group and there were changes in plasma and urinary concentrations of several of the sulfur amino acids and in adipose tissue mRNA expression of particularly *DGAT1* in the Met/Cys_-low_ group. The Met/Cys_-low_ and Met/Cys_-medium_ groups tended towards reductions in plasma SCD1 activity index. The findings on adipose tissue mRNA expression of *DGAT1* and *ACAC* as well as SCD1 activity indices in plasma became more pronounced when we excluded one outlier with high HOMA-IR at baseline.

The increases in serum FGF21 in the Met/Cys_-low_ group is in line with preclinical studies showing upregulated hepatic gene expression and elevated circulating FGF21 in response to methionine restriction [14, 18, 39]. The range of serum FGF21 concentrations was wide and may have introduced some bias in our analyses, however, our findings also agree with a human study (n = 72) that showed increased plasma FGF21 concentrations in vegans and vegetarians compared with omnivores, and that plasma FGF21 concentrations increased significantly in omnivore subjects (n = 16) given a vegetarian diet for 4 d [40]. Many plant foods in vegetarian diets are naturally low in sulfur amino acid, whereas certain fruits, vegetables and grains have higher content of methionine and cysteine. Our study reports for the first-time changes in serum FGF21 in a randomized, controlled study testing a strict vegan-based methionine and cysteine restricted diet. Our findings may indicate that the metabolic response in humans is similar as demonstrated in rodents. Whether methionine and cysteine restriction through a strict vegan diet or more moderately restrictive vegetarian diets may provide metabolic benefits via increased circulating levels of FGF21 needs further investigation.

In line with our previously published 7-days pilot study in normal-weight individuals [24], we observed decreased plasma concentrations of methionine and cystathionine, and increased plasma total homocysteine in the Met/Cys_-low_ group as compared with the Met/Cys_-high_ group. In our previous pilot study, normal-weight subjects exhibited a slight increase in the cysteine–cysteine disulphide, cystine [24]. In the present study we observed a reduction in cystine in the Met/Cys_-low_ group compared with the Met/Cys_-high_ group, which were in line with a previous 16-week study in obese participants [22]. The contrasting plasma cystine responses between our present and previous pilot studies [24] might be due to differences in body adiposity. Baseline concentrations of cystine in the participants with overweight and obesity in our current study were somewhat higher than in the participants with normal-weight (mean of 44 versus 36 µmol/L). The increase in plasma cystine in the previous pilot study with normal-weight individuals was suggested to be due to compensatory mechanisms, including decline in the urinary excretion of tCys, and potential activation of *CDO*, which is the rate-limiting enzyme in oxidation of cysteine to taurine [33]. Although we did observe similar decreases in urinary tCys in our present study, we did not observe changes in expression of *CDO* in adipose tissue. This was not surprising as *CDO* is regulated at the protein level, and is mostly expressed in the liver [33]. Overall, the cause-effect of this association is still unclear, but this observation reinforces the hypothesis that metabolism of cysteine may be implicated in development of obesity.

Glutathione can serve as a reservoir for cysteine during periods of deficient intakes [41]. However, we observed that expression of *GCLM,* which is crucial in glutathione synthesis, tended to be enhanced in the Met/Cys_-low_ compared to the Met/Cys_-high_ group. The clear trend for an increased expression of *GCLM* mRNA during low dietary cysteine intakes may seem paradoxical. However, a previous study demonstrated similar findings in mice, suggesting that cysteine deprivation may enhance glutathione production to protect against cellular stress, at least in the short term [31]. We do not have sufficient data to draw conclusions on glutathione production, and note that no changes were observed between groups in plasma glutathione fractions. The implication of these findings will have to be explored in future human and experimental studies.

We observed effects on subcutaneous adipose tissue *DGAT1* mRNA expression in the Met/Cys_-low_ group, and a tendency towards induction of other genes involved in lipid metabolism (including *DGAT1*, *ACAC*, *SCD1*, and *CPT1A*). The effects became more pronounced for most lipid-related transcripts when we excluded a subject with high baseline HOMA-IR. Our findings are consistent with preclinical data, where dietary methionine restriction upregulates enzymes involved in lipogenesis, triglyceride synthesis and fatty acid oxidation [16, 32, 42, 43]. The simultaneous increase of both lipogenesis and fatty acid oxidation in methionine restricted rodents, points to a futile cycle of lipid metabolism in subcutaneous adipose tissue [13, 16]. However, rodent studies have shown that the hepatic mRNA expression pattern is somewhat different than that in subcutaneous adipose tissue, with reduced expression of transcripts in lipid synthesis (e.g., *SCD1, DGAT1*), promoting a shift from fatty acid synthesis to fatty acid oxidation in the liver [13, 17, 32]. It seems plausible that it is not merely changes in adipose tissue or liver that explains the beneficial effects of methionine and cysteine restriction in rodents, but tissue-specific changes might reflect whole-body changes in lipid turnover contributing to effects on overall energy metabolism.

The present study included one participant with high HOMA-IR at baseline. Although initially included in the main analyses, the participant was excluded from analyses investigating the effect of the intervention on subcutaneous adipose tissue gene expression and the SCD-16 index (surrogate of hepatic SCD1 activity). When we excluded the participant from these analyses, several findings appeared stronger including expression of genes involved in lipogenesis as well as the plasma SCD-16 index. The responses in subcutaneous adipose tissue mRNA expression and the reduction in plasma SCD-16 index after exclusion appear to be consistent with preclinical studies [13, 17, 32]. This potential confounding effect of insulin on relevant outcomes should be taken into account when designing dietary intervention studies examining the effect of dietary methionine and cysteine restriction.

This is the first study reporting findings from a combined dietary methionine and cysteine restriction in humans. The main strength of this study is the randomized, controlled and double-blind design which is rare for dietary interventions, and that we were able to design diets that only differed by methionine and cysteine content and were otherwise identical. Another major strength is the translational approach and that the study provides valuable insight for future large-scale studies investigating whether the beneficial findings of methionine and cysteine restriction in animals also pertain to humans. In particular, we have collected and reported data on a wide range of outcomes that have previously been identified in preclinical as well as epidemiological studies, including plasma concentrations of amino acids [8], expression of subcutaneous adipose tissue genes involved in lipid metabolism [44], plasma fatty acid desaturase indices [9, 13] as well as plasma parameters related to lipid and glucose metabolism [45, 46]. In addition, we have evaluated compliance and feasibility of the diet, which may be a concern with vegan-based interventions. The main limitation is the small sample size, and consequently low power, but considering that this was a pilot study, no power calculations were performed prior to study commencement. The small sample size contributes to substantial variation seen in the response to the diet in most markers and it is clear that larger and longer studies are warranted to further establish the observed relationships. In addition, there were some differences in baseline characteristics such as age, BMI and serum triglycerides, which may be due to unfortunate randomization. When we adjusted outcome analyses for these imbalances, results were not altered materially. The duration was short, and whether the effects we observed are sustained over time is unknown. Finally, the content of methionine and cysteine in the Met/Cys_-medium_ group may have been too high to achieve the same beneficial effects as the Met/Cys_-low_ group as seen in moderate restriction in animal models [47]. We note that the results reported are meant to aid in the design and implementation of future large-scale studies, and we cannot draw strong conclusions about the effect(s) of methionine and cysteine restriction in humans as per CONSORT statements for reporting pilot trials. Several of the findings that were statistically non-significant—but tended clearly in one direction and were in line with findings from preclinical studies—will be considered potential outcomes in future studies.

## Conclusions

Results from this pilot trial with diets that differed only by methionine and cysteine content indicate that such an intervention is feasible in humans and might have effects that are comparable to demonstrations in animals. Due to the small sample size and short duration these results must be interpreted with care, but merit future, long-term investigations into whether dietary methionine and cysteine restriction can confer sustainable and beneficial effects over time in overweight and obese populations. Because the Met/Cys_-medium_ followed similar patterns as the Met/Cys_-high_ group with respect to most outcomes, a diet low in methionine and cysteine should be utilized to maximize effects in future studies.

## Supplementary information

**Additional file 1.** Mean nutrient content and composition in the 7-day diet, excluding SAA-powder.

**Additional file 2.** A typical daily menu in the 7-day diet.

**Additional file 3.** Mean daily sulfur amino acid intake in the low, moderate and high Met/Cys diets.

**Additional file 4.** Primer sequences of gene expression measured by qPCR.

**Additional file 5.** Spearman’s rank correlation coefficients and 95% confidence intervals between sulfur amino acids and related intermediates.

**Additional file 6.** Estimated means linear predictions/responses in plasma sulfur amino acids. Values are derived from a linear mixed model regression. The p-values denote the p for interaction between group and time and indicate the difference in response over time between the Met/Cys_-medium_ and Met/Cys_-high_ groups. Abbreviations: Met/Cys, methionine and cysteine; SAM, S-adenosylmethionine, SAH, S-adenosylhomocysteine; GSH, glutathione; GSSG, oxidized glutathione.

**Additional file 7.** Estimated means linear predictions/response in creatinine-adjusted urinary sulfur amino acid concentrations. Values are derived from a linear mixed model regression. The p-values denote the p for interaction between group and time and indicate the difference in response over time between the Met/Cys_-low_ and Met/Cys_-high_ groups. Abbreviations: Met/Cys, methionine and cysteine.

**Additional file 8.** Estimated means linear predictions/response in creatinine-adjusted urinary sulfur amino acid concentrations. Values are derived from a linear mixed model regression. The p-values denote the p for interaction between group and time and indicate the difference in response over time between the Met/Cys_-medium_ and Met/Cys_-high_ groups. Abbreviations: Met/Cys, methionine and cysteine.

**Additional file 9.** Estimated means linear predictions/response in serum Fibroblast growth Factor 21 to the diets. Values are derived from a linear mixed model regression. The p-values denote the p for interaction between group and time and indicate the difference in response over time between the Met/Cys_-medium_ and Met/Cys_-high_ groups. Abbreviations: FGF21, fibroblast growth factor 21; Met/Cys, methionine and cysteine.

**Additional file 10.** Individual responses in mRNA expression in subcutaneous adipose tissue in the Met/Cys_-low_ group. Abbreviations; Met/Cys, methionine and cysteine; MTR, methionine synthase reductase; CBS, cystathionine-β-synthase; CDO, cysteine dioxygenase; GCLC, glutamate-cysteine ligase catalytic subunit; GCLM, glutamate-cysteine ligase regulatory subunit; CPT1A, carnitine palmitoyl-transferase 1a; ACACA, acetyl-CoA carboxylase; FASN, fatty acid synthase; DGAT1, diacylglycerol *O*-acyltransferase 1; SCD1, stearoyl CoA-desaturase 1; SREBP, sterol regulatory element binding protein; PPARG, peroxisome-proliferator activated receptor γ; MTOR, mammalian target of rapamycin; LEP, leptin; PRDM16, PR domain containing 16.

**Additional file 11.** Individual responses in mRNA expression in subcutaneous adipose tissue in the Met/Cys_-medium_ group. Abbreviations; Met/Cys, methionine and cysteine; MTR, methionine synthase reductase; CBS, cystathionine-β-synthase; CDO, cysteine dioxygenase; GCLC, glutamate-cysteine ligase catalytic subunit; GCLM, glutamate-cysteine ligase regulatory subunit; CPT1A, carnitine palmitoyl-transferase 1a; ACACA, acetyl-CoA carboxylase; FASN, fatty acid synthase; DGAT1, diacylglycerol *O*-acyltransferase 1; SCD1, stearoyl CoA-desaturase 1; SREBP, sterol regulatory element binding protein; PPARG, peroxisome-proliferator activated receptor γ; MTOR, mammalian target of rapamycin; LEP, leptin; PRDM16, PR domain containing 16.

**Additional file 12.** Individual responses in mRNA expression in subcutaneous adipose tissue in the Met/Cys_-high_ group. Abbreviations; Met/Cys, methionine and cysteine; MTR, methionine synthase reductase; CBS, cystathionine-β-synthase; CDO, cysteine dioxygenase; GCLC, glutamate-cysteine ligase catalytic subunit; GCLM, glutamate-cysteine ligase regulatory subunit; CPT1A, carnitine palmitoyl-transferase 1a; ACACA, acetyl-CoA carboxylase; FASN, fatty acid synthase; DGAT1, diacylglycerol *O*-acyltransferase 1; SCD1, stearoyl CoA-desaturase 1; SREBP, sterol regulatory element binding protein; PPARG, peroxisome-proliferator activated receptor γ; MTOR, mammalian target of rapamycin; LEP, leptin; PRDM16, PR domain containing 16.

**Additional file 13.** Correlation (95% confidence interval) between changes in plasma sulfur amino acids and gene expression.

## Data Availability

The datasets used and/or analysed during the current study are available from the corresponding author on reasonable request.

## Abbreviations

ACAC: Acetyl-CoA carboxylase
apoA: Apolipoprotein A
apoB: Apolipoprotein B
BMI: Body mass index
CDO: Cysteine dioxygenase
FASN: Fatty acid synthase
GCLC: Glutamate-cysteine ligase catalytic subunit
GCLM: Glutamate-cysteine ligase modifier subunit
CPT1A: Carnitine palmitoyltransferase I
DGAT1: Diacylglycerol O-Acyltransferase 1
HDL-C: High-density lipoprotein cholesterol
HOMA-IR: Homeostatic model of insulin resistance
LDL-C: Low-density lipoprotein cholesterol, MTOR, mammalian target of rapamycin
tCys: Total cysteine
PRDM16: PR/SET domain 16
SCD: Stearoyl coenzyme A desaturase
SREBP1: Sterol regulatory element-binding protein 1
