# Supplementary material for: Effects of dietary methionine and cysteine restriction on plasma biomarkers, serum fibroblast growth factor 21, and adipose tissue gene expression in women with overweight or obesity: a double-blind randomized controlled pilot study
Source: J Transl Med. 2020 Mar 11;18:122. doi: 10.1186/s12967-020-02288-x (PMC7065370; doi:10.1186/s12967-020-02288-x)
Supplement: Supplementary file 1 — Additional file 1. Mean nutrient content and composition in the 7-day diet, excluding SAA-powder. [file 12967_2020_2288_MOESM1_ESM.docx]

| **Additional file 1. Mean nutrient content and composition in the 7-day diet, excluding SAA-powder.** | |
| --- | --- |
| Kcal | 2190.0 |
| Fat, % of energy | 30.2 |
| Fat, g | 73.4 |
| SFA, % of energy | 3.9 |
| SFA, g | 9.5 |
| MUFA, % of energy | 16.2 |
| MUFA, g | 39.3 |
| PUFA, % of energy | 7.2 |
| PUFA, g | 17.6 |
| n-3 PUFA, g | 2.6 |
| n-6 PUFA, g | 13.4 |
| Carbohydrates, % of energy | 48.3 |
| Carbohydrates, g | 264.2 |
| Proteins, % of energy | 15.7 |
| Proteins, g | 86.1 |
| Sulfur amino acids, g | 1.6 |
| Methionine, g | 0.8 |
| Cysteine, g | 0.8 |
| Numbers expressed as mean. SAAs: Sulfur amino acids; SFA: saturated fatty acids; MUFA: monounsaturated fatty acids; PUFA: polyunsaturated fatty acids. | |
