# Supplementary material for: Effects of dietary methionine and cysteine restriction on plasma biomarkers, serum fibroblast growth factor 21, and adipose tissue gene expression in women with overweight or obesity: a double-blind randomized controlled pilot study
Source: J Transl Med. 2020 Mar 11;18:122. doi: 10.1186/s12967-020-02288-x (PMC7065370; doi:10.1186/s12967-020-02288-x)
Supplement: Supplementary file 2 — Additional file 2. A typical daily menu in the 7-day diet. [file 12967_2020_2288_MOESM2_ESM.docx]

| **Additional file 2**. A typical daily menu in the 7-day diet | | |
| --- | --- | --- |
| Breakfast | Oat meal with apple/raisins and cinnamon | |
| Lunch | Salad with beans, fruits and vegetables and vegetable oil | |
| Dinner | Vegetable casserole/vegetable soup/bean salad | |
| Supper | Vegetable soup with bread/focaccia | |
| *Supplemental protein drinks (with or without methionine and cysteine) with each meal* | | |
| Snack | | Nuts/fruits |
| Vegan-based diets without meat, fish, eggs, dairy, and certain plant-based foods. The diets differed only by methionine and cysteine content through supplemental protein drinks. | | |
