# Supplementary material for: Effects of dietary methionine and cysteine restriction on plasma biomarkers, serum fibroblast growth factor 21, and adipose tissue gene expression in women with overweight or obesity: a double-blind randomized controlled pilot study
Source: J Transl Med. 2020 Mar 11;18:122. doi: 10.1186/s12967-020-02288-x (PMC7065370; doi:10.1186/s12967-020-02288-x)
Supplement: Supplementary file 3 — Additional file 3. Mean daily sulfur amino acid intake in the low, moderate and high Met/Cys diets. [file 12967_2020_2288_MOESM3_ESM.docx]

| **Additional file 3. Mean daily sulfur amino acid intake in the low, moderate and high Met/Cys diets.** | | | | | | |
| --- | --- | --- | --- | --- | --- | --- |
|  | Met/Cys_-low_ | | Met/Cys_-medium_ | | Met/Cys_-high_ | |
|  | Diet / Supplement | Total | Diet / Supplement | Total | Diet / Supplement | Total |
| Sulfur amino acids, g | 1.6 / 0.0 | 1.6 | 1.6 / 2.04 | 3.64 | 1.6 / 4.0 | 5.6 |
| Methionine, g | 0.8 / 0.0 | 0.8 | 0.8 / 0.68 | 1.48 | 0.8 / 1.32 | 2.12 |
| Cysteine, g | 0.8 / 0.0 | 0.8 | 0.8 / 1.36 | 2.16 | 0.8 / 2.68 | 3.48 |
| Met/Cys_-low_: diet low in methionine and cysteine. Met/Cys_-medium_: diet moderate in methionine and cysteine. Met/Cys_-high_: diet high in methionine and cysteine. | | | | | | |
