# Supplementary material for: Effects of dietary methionine and cysteine restriction on plasma biomarkers, serum fibroblast growth factor 21, and adipose tissue gene expression in women with overweight or obesity: a double-blind randomized controlled pilot study
Source: J Transl Med. 2020 Mar 11;18:122. doi: 10.1186/s12967-020-02288-x (PMC7065370; doi:10.1186/s12967-020-02288-x)
Supplement: Supplementary file 4 — Additional file 4. Primer sequences of gene expression measured by qPCR. [file 12967_2020_2288_MOESM4_ESM.docx]

| **Additional file 4. Primer sequences of gene expression measured by qPCR** | |
| --- | --- |
| Primer | Sequence |
| *TBP* | GTGGGGAGCTGTGATGTGAA  ACCAGGAAATAACTCTGGCTCA |
| *MTOR* | \| TGGGGTTTAGGTCAGTGGGA \| \| --- \| \| CAGTGCCAGCACAGCTCTAT \| |
| *MTR* | \| AACAGTAGGTGGAGAGGCCA \| \| --- \| \| GGGCCCGGAGTTTCTTTTGA \| |
| *ACACA* | \| ATTGGGGCTTACCTTGTCCG \| \| --- \| \| CGAGGACTTTGTTGAGGGCT \| |
| *DGAT1* | \| AGCCCTTCAAGGACATGGAC \| \| --- \| \| GATGAGGTGATTGGGGACCG \| |
| *GCLC* | \| GATTGTCGCTGGGGAGTGAT \| \| --- \| \| TCTTCAATGGCTCCAGTCCTC \| |
| *SREBP* | \| CCGCTCCTCCATCAATGACA \| \| --- \| \| GCTGTGTTGCAGAAAGCGAA \| |
| *CPT1A* | \| ATGTACGCCAAGATCGACCC \| \| --- \| \| GACATGCAGTTGGCCGTTTC \| |
| *CBS* | \| GAGAAGAAGCCCTGGTGGTG \| \| --- \| \| CGGAGGATCTCGATGGTGTG \| |
| *CDO* | \| AACTCCAAATGCAACTTCGGG \| \| --- \| \| CCAAGGCAAACATACAGCGA \| |
| *SCD1* | \| CTGCAGGACGATATCTCTAGCTC \| \| --- \| \| TCCAAGTAGAGGGGCATCGT \| |
| *PPARG* | \| ACAGATCCAGTGGTTGCAGA \| \| --- \| \| TCCACTTTGATTGCACTTTGGT \| |
| *GCLC* | \| GTGGGCACAGGTAAAACCAAA \| \| --- \| \| CAAATCTGGTGGCATCACACA \| |
| *FASN* | \| CTTCAAGGAGCAAGGCGTGA \| \| --- \| \| ACTGGTACAACGAGCGGATG \| |
