# Supplementary figures and images for: Effects of dietary methionine and cysteine restriction on plasma biomarkers, serum fibroblast growth factor 21, and adipose tissue gene expression in women with overweight or obesity: a double-blind randomized controlled pilot study
Source: J Transl Med. 2020 Mar 11;18:122. doi: 10.1186/s12967-020-02288-x (PMC7065370; doi:10.1186/s12967-020-02288-x)

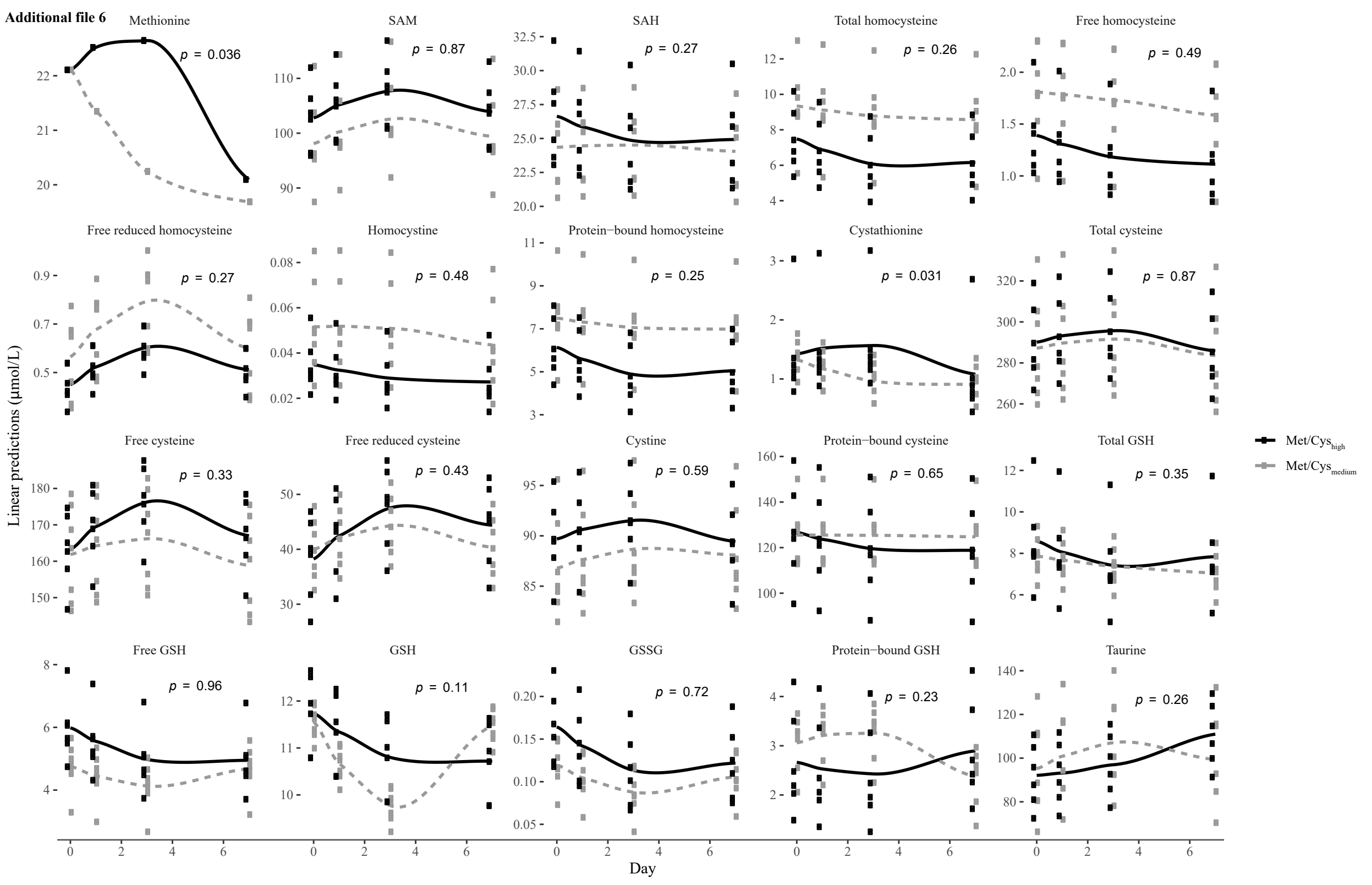

Supplement: Supplementary file 6 — Additional file 6. Estimated means linear predictions/responses in plasma sulfur amino acids. Values are derived from a linear mixed model regression. The p-values denote the p for interaction between group and time and indicate the difference in response over time between the Met/Cys-medium and Met/Cys-high groups. Abbreviations: Met/Cys, methionine and cysteine; SAM, S-adenosylmethionine, SAH, S-adenosylhomocysteine; GSH, glutathione; GSSG, oxidized glutathione. [file 12967_2020_2288_MOESM6_ESM.pdf]

Additional file 8

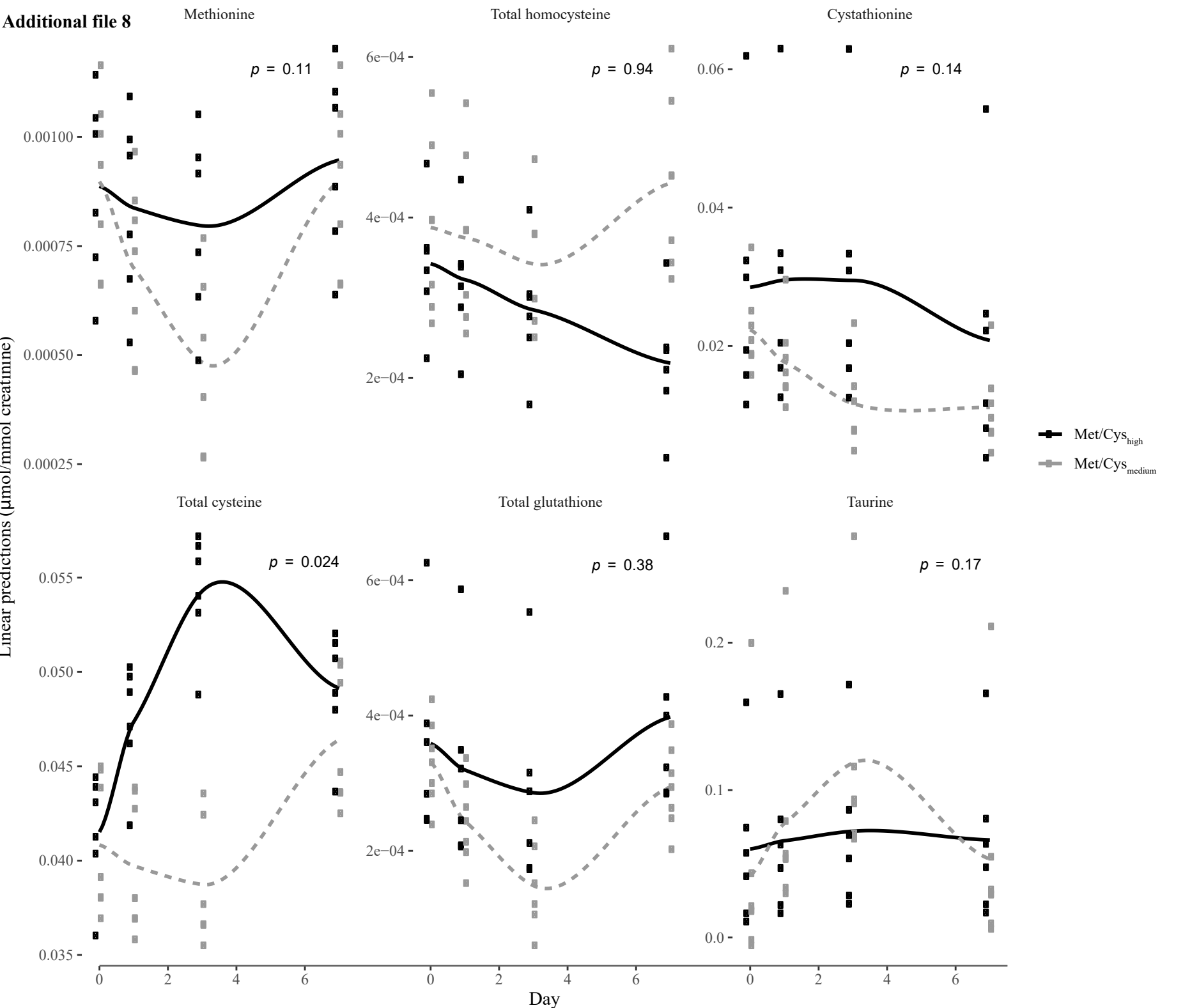

Supplement: Supplementary file 8 — Additional file 8. Estimated means linear predictions/response in creatinine-adjusted urinary sulfur amino acid concentrations. Values are derived from a linear mixed model regression. The p-values denote the p for interaction between group and time and indicate the difference in response over time between the Met/Cys-medium and Met/Cys-high groups. Abbreviations: Met/Cys, methionine and cysteine. [file 12967_2020_2288_MOESM8_ESM.pdf]

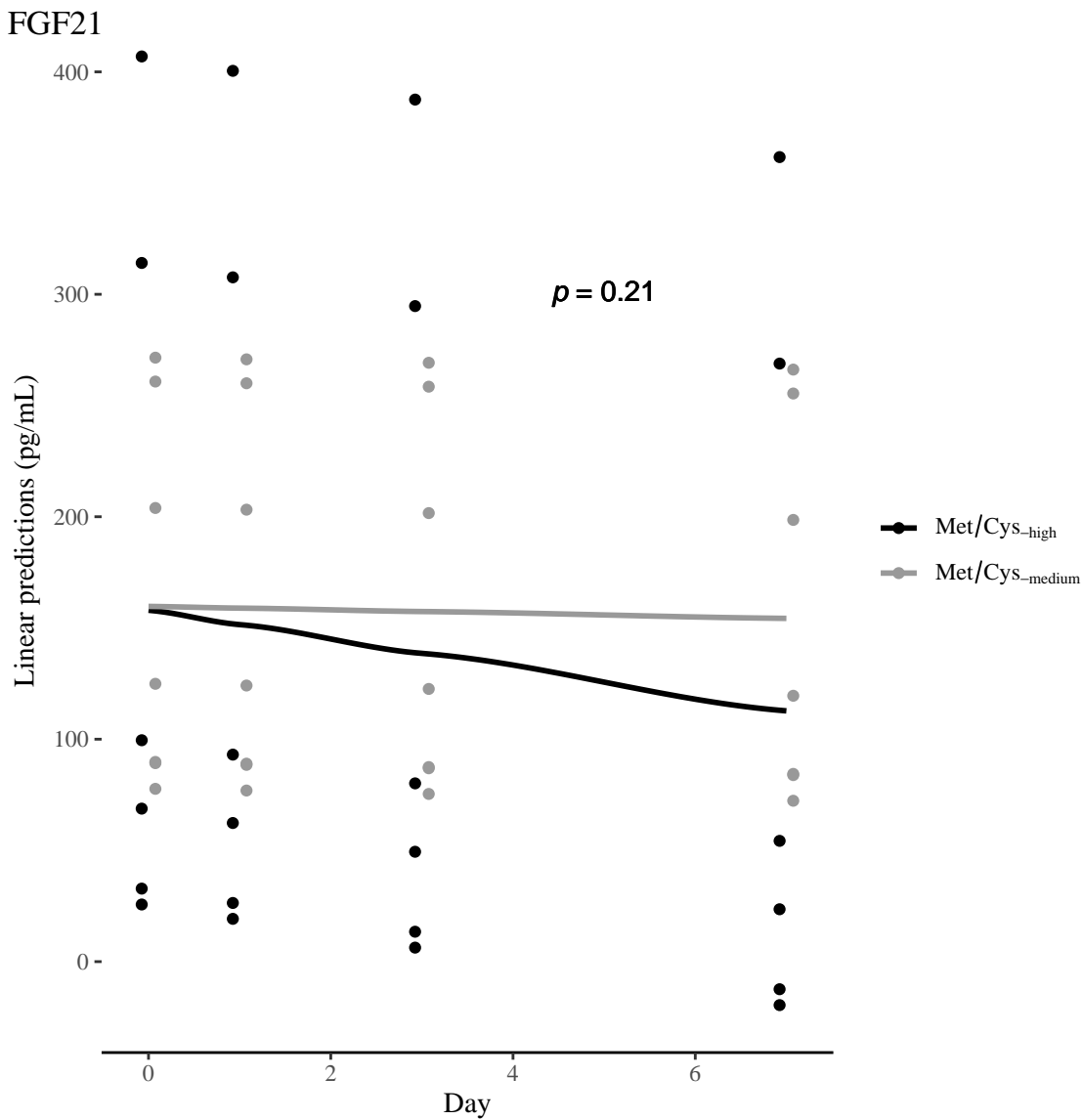

Supplement: Supplementary file 9 — Additional file 9. Estimated means linear predictions/response in serum Fibroblast growth Factor 21 to the diets. Values are derived from a linear mixed model regression. The p-values denote the p for interaction between group and time and indicate the difference in response over time between the Met/Cys-medium and Met/Cys-high groups. Abbreviations: FGF21, fibroblast growth factor 21; Met/Cys, methionine and cysteine. [file 12967_2020_2288_MOESM9_ESM.pdf]

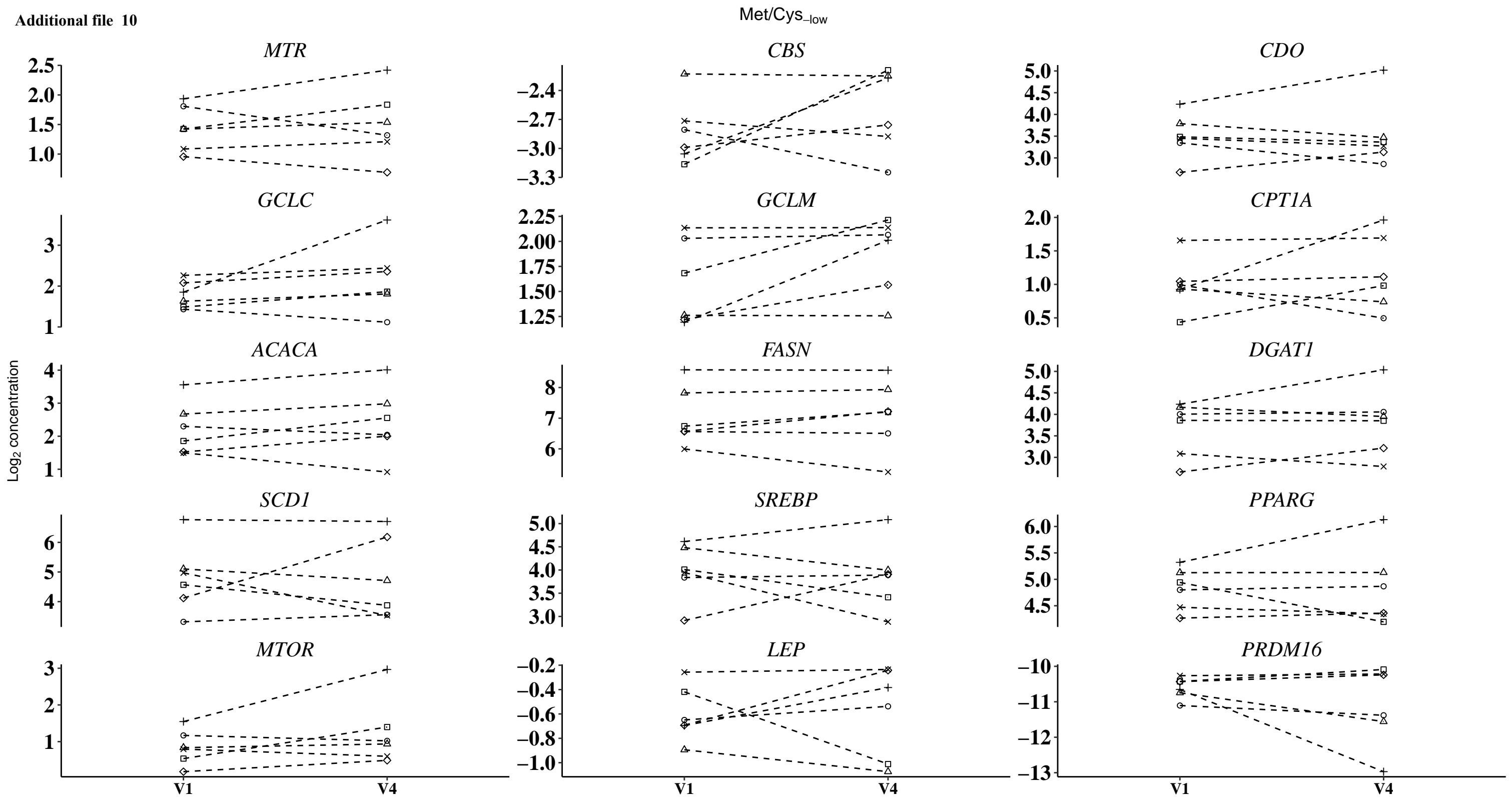

Supplement: Supplementary file 10 — Additional file 10. Individual responses in mRNA expression in subcutaneous adipose tissue in the Met/Cys-low group. Abbreviations; Met/Cys, methionine and cysteine; MTR, methionine synthase reductase; CBS, cystathionine-β-synthase; CDO, cysteine dioxygenase; GCLC, glutamate-cysteine ligase catalytic subunit; GCLM, glutamate-cysteine ligase regulatory subunit; CPT1A, carnitine palmitoyl-transferase 1a; ACACA, acetyl-CoA carboxylase; FASN, fatty acid synthase; DGAT1, diacylglycerol O-acyltransferase 1; SCD1, stearoyl CoA-desaturase 1; SREBP, sterol regulatory element binding protein; PPARG, peroxisome-proliferator activated receptor γ; MTOR, mammalian target of rapamycin; LEP, leptin; PRDM16, PR domain containing 16. [file 12967_2020_2288_MOESM10_ESM.pdf]

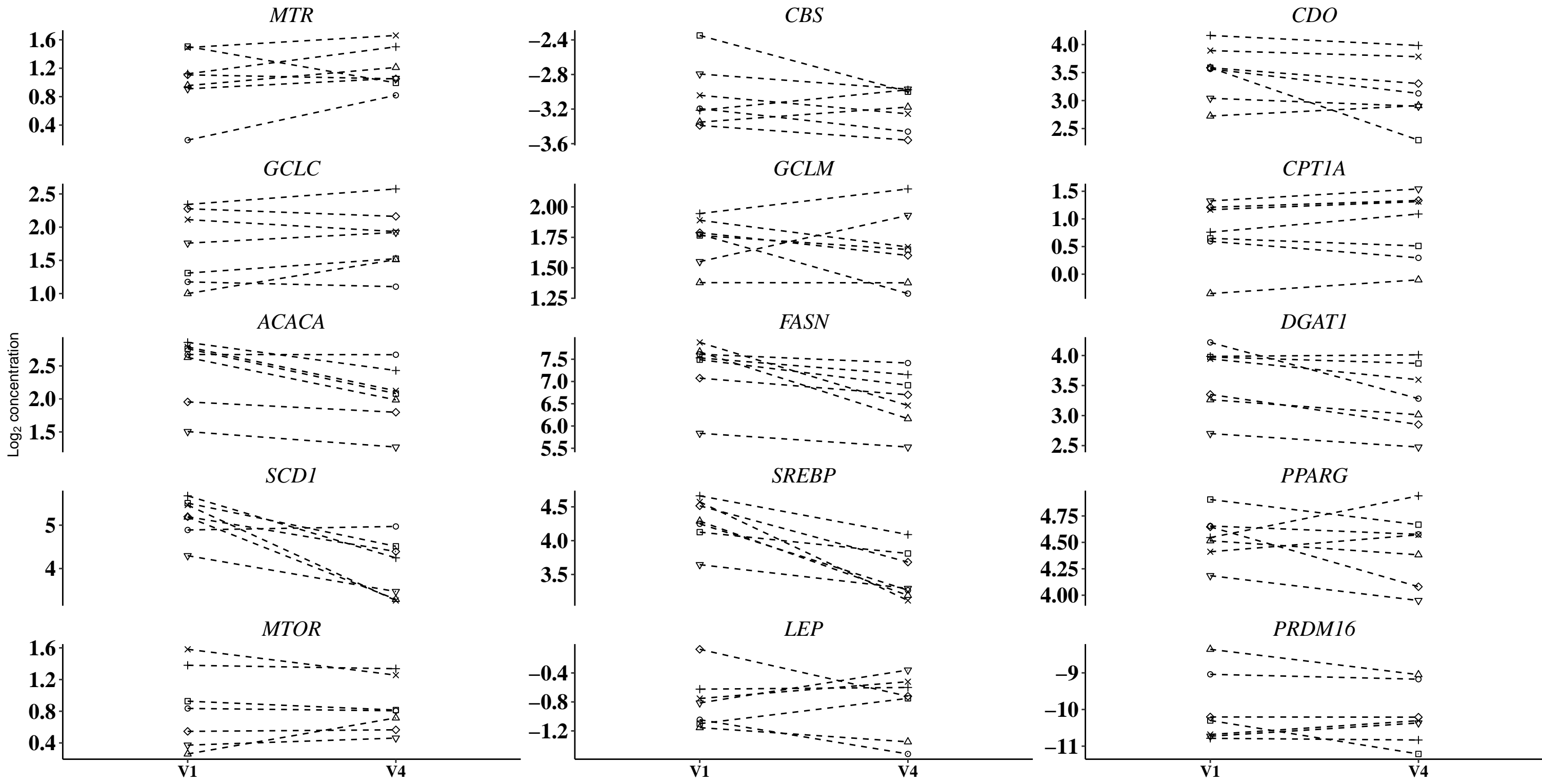

Supplement: Supplementary file 11 — Additional file 11. Individual responses in mRNA expression in subcutaneous adipose tissue in the Met/Cys-medium group. Abbreviations; Met/Cys, methionine and cysteine; MTR, methionine synthase reductase; CBS, cystathionine-β-synthase; CDO, cysteine dioxygenase; GCLC, glutamate-cysteine ligase catalytic subunit; GCLM, glutamate-cysteine ligase regulatory subunit; CPT1A, carnitine palmitoyl-transferase 1a; ACACA, acetyl-CoA carboxylase; FASN, fatty acid synthase; DGAT1, diacylglycerol O-acyltransferase 1; SCD1, stearoyl CoA-desaturase 1; SREBP, sterol regulatory element binding protein; PPARG, peroxisome-proliferator activated receptor γ; MTOR, mammalian target of rapamycin; LEP, leptin; PRDM16, PR domain containing 16. [file 12967_2020_2288_MOESM11_ESM.pdf]

Met/Cys<sub>high</sub>

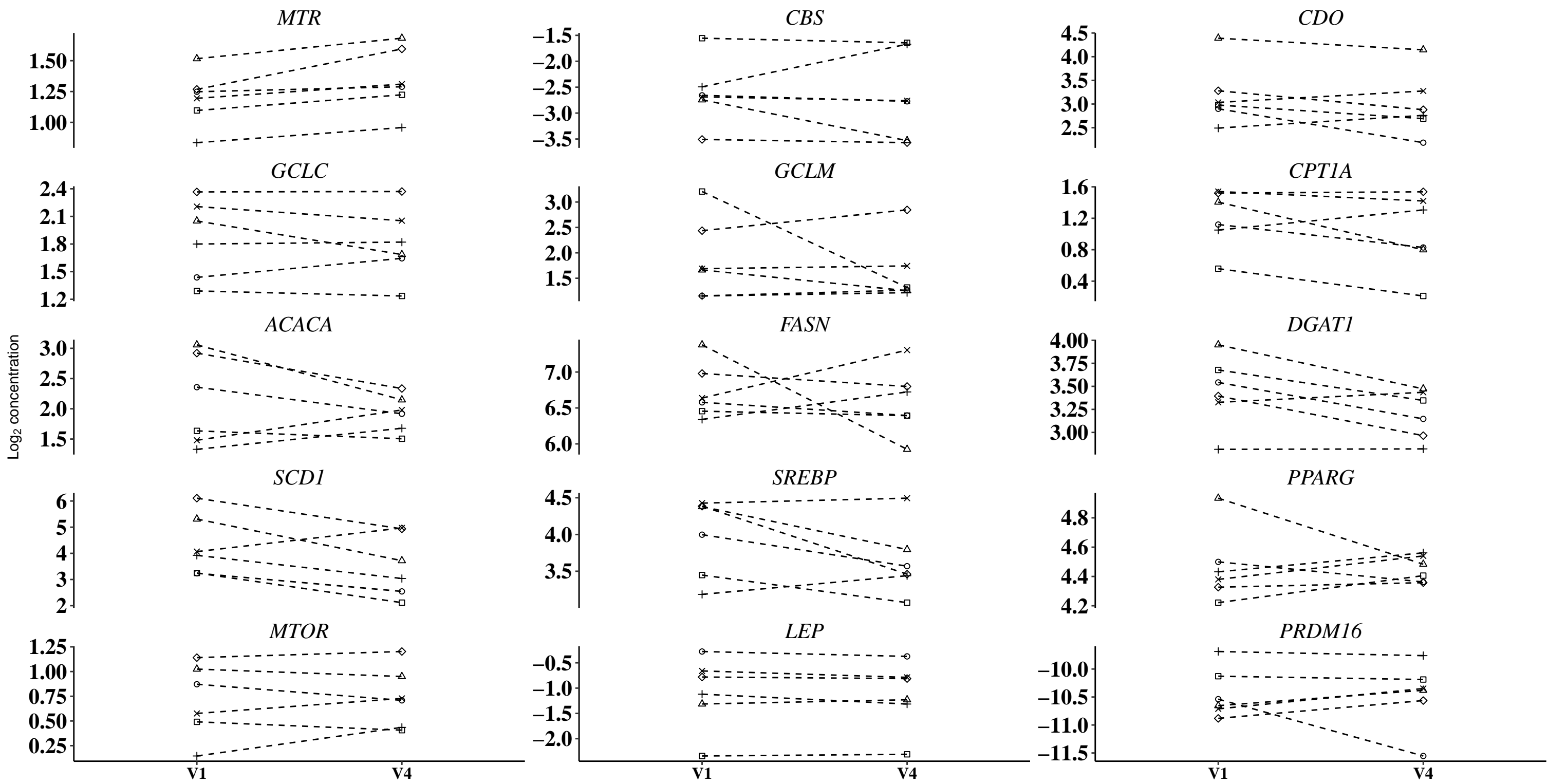

Supplement: Supplementary file 12 — Additional file 12. Individual responses in mRNA expression in subcutaneous adipose tissue in the Met/Cys-high group. Abbreviations; Met/Cys, methionine and cysteine; MTR, methionine synthase reductase; CBS, cystathionine-β-synthase; CDO, cysteine dioxygenase; GCLC, glutamate-cysteine ligase catalytic subunit; GCLM, glutamate-cysteine ligase regulatory subunit; CPT1A, carnitine palmitoyl-transferase 1a; ACACA, acetyl-CoA carboxylase; FASN, fatty acid synthase; DGAT1, diacylglycerol O-acyltransferase 1; SCD1, stearoyl CoA-desaturase 1; SREBP, sterol regulatory element binding protein; PPARG, peroxisome-proliferator activated receptor γ; MTOR, mammalian target of rapamycin; LEP, leptin; PRDM16, PR domain containing 16. [file 12967_2020_2288_MOESM12_ESM.pdf]
