# Supplementary material for: Effects of dietary methionine and cysteine restriction on plasma biomarkers, serum fibroblast growth factor 21, and adipose tissue gene expression in women with overweight or obesity: a double-blind randomized controlled pilot study
Source: J Transl Med. 2020 Mar 11;18:122. doi: 10.1186/s12967-020-02288-x (PMC7065370; doi:10.1186/s12967-020-02288-x)

**Additional file 7**

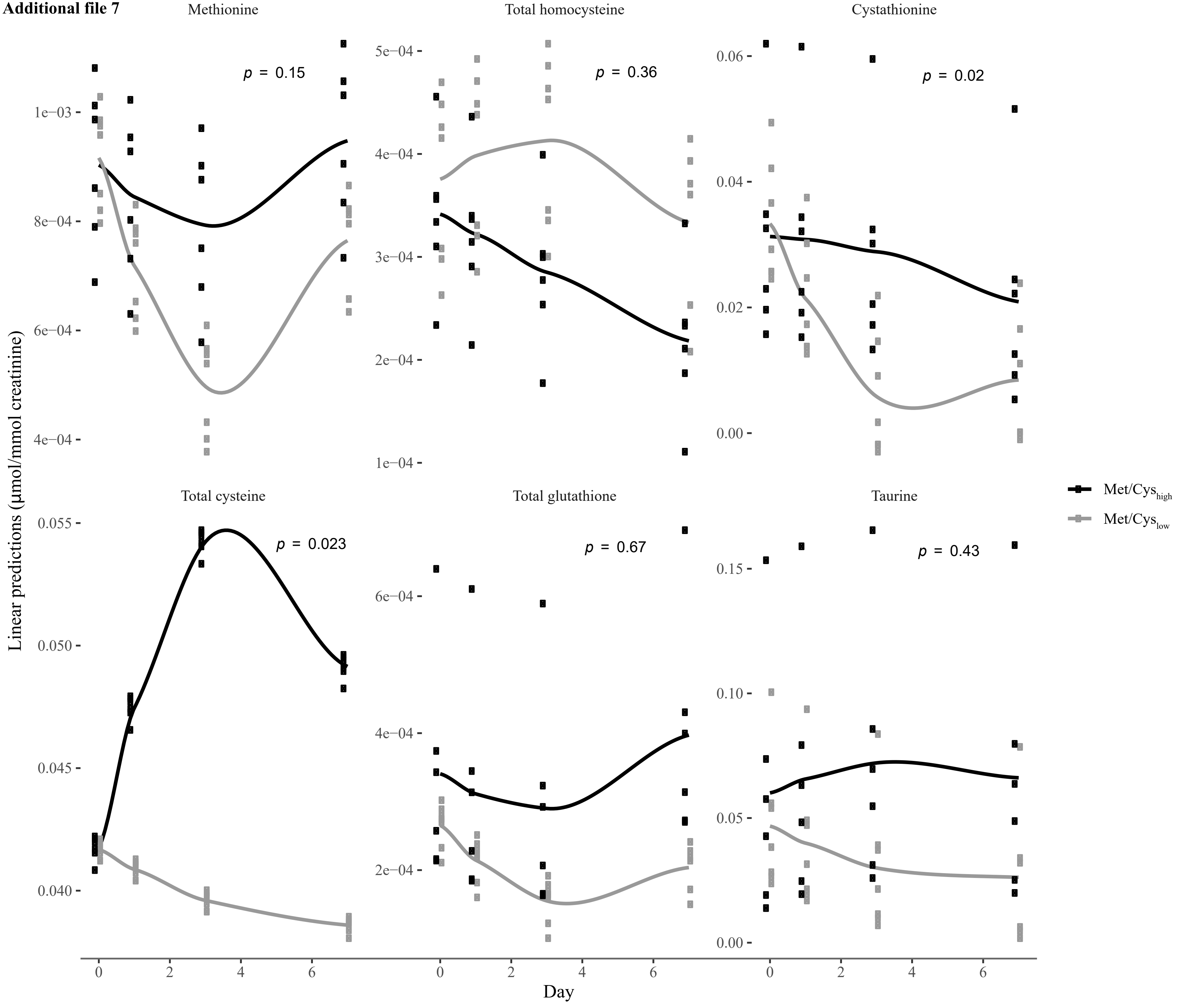

Supplement: Supplementary file 7 — Additional file 7. Estimated means linear predictions/response in creatinine-adjusted urinary sulfur amino acid concentrations. Values are derived from a linear mixed model regression. The p-values denote the p for interaction between group and time and indicate the difference in response over time between the Met/Cys-low and Met/Cys-high groups. Abbreviations: Met/Cys, methionine and cysteine. [file 12967_2020_2288_MOESM7_ESM.pdf]
