# Supplementary material for: Effects of dietary methionine and cysteine restriction on plasma biomarkers, serum fibroblast growth factor 21, and adipose tissue gene expression in women with overweight or obesity: a double-blind randomized controlled pilot study
Source: J Transl Med. 2020 Mar 11;18:122. doi: 10.1186/s12967-020-02288-x (PMC7065370; doi:10.1186/s12967-020-02288-x)
Supplement: Supplementary file 13 — Additional file 13. Correlation (95% confidence interval) between changes in plasma sulfur amino acids and gene expression. [file 12967_2020_2288_MOESM13_ESM.docx]

| **Additional file 13. Correlation (95 % confidence interval) between changes in plasma sulfur amino acids and gene expression** | | | | | | | | | | | | | | | | | |
| --- | --- | --- | --- | --- | --- | --- | --- | --- | --- | --- | --- | --- | --- | --- | --- | --- | --- |
|  | ACACA | CBS | CDO | CPT1A | DGAT1 | FASN | GCLC | GCLM | LEP | MTOR | MTR | PPARG | PRDM16 | SCD1 | SREBP | UCP1 |  |
| Methionine | -0.14 (-0.56, 0.33) | 0.15 (-0.33, 0.56) | 0.26 (-0.22, 0.64) | 0.15 (-0.32, 0.57) | -0.19 (-0.59, 0.29) | -0.098 (-0.53, 0.37) | -0.2 (-0.6, 0.28) | 0.14 (-0.33, 0.56) | -0.061 (-0.5, 0.4) | -0.0035 (-0.46, 0.45) | 0.19 (-0.28, 0.6) | 0.14 (-0.33, 0.56) | 0.31 (-0.17, 0.67) | -0.33 (-0.68, 0.15) | 0.007 (-0.45, 0.46) | 0.19 (-0.29, 0.59) |  |
| SAM | 0.084 (-0.38, 0.52) | -0.018 (-0.47, 0.44) | -0.06 (-0.5, 0.41) | -0.023 (-0.47, 0.44) | 0.12 (-0.35, 0.55) | 0.23 (-0.25, 0.62) | -0.22 (-0.61, 0.26) | 0.3 (-0.17, 0.67) | 0.21 (-0.27, 0.61) | -0.072 (-0.51, 0.4) | -0.081 (-0.52, 0.39) | -0.007 (-0.46, 0.45) | -0.037 (-0.48, 0.42) | 0.11 (-0.36, 0.54) | 0.34 (-0.14, 0.69) | -0.2 (-0.6, 0.28) |  |
| SAH | -0.035 (-0.48, 0.43) | -0.081 (-0.52, 0.39) | -0.018 (-0.47, 0.44) | -0.046 (-0.49, 0.42) | 0.24 (-0.24, 0.62) | 0.042 (-0.42, 0.49) | -0.29 (-0.66, 0.18) | -0.0088 (-0.46, 0.45) | 0.29 (-0.19, 0.66) | -0.23 (-0.62, 0.25) | 0.037 (-0.42, 0.48) | 0.19 (-0.28, 0.6) | 0.19 (-0.29, 0.59) | -0.19 (-0.6, 0.28) | 0.19 (-0.28, 0.6) | -0.28 (-0.65, 0.2) |  |
| Total homocysteine | 0.2 (-0.28, 0.6) | 0.13 (-0.34, 0.55) | 0.28 (-0.2, 0.65) | 0.38 (-0.091, 0.71) | 0.36 (-0.11, 0.7) | 0.011 (-0.45, 0.46) | 0.29 (-0.19, 0.66) | 0.25 (-0.23, 0.63) | -0.03 (-0.48, 0.43) | 0.26 (-0.22, 0.64) | 0.34 (-0.13, 0.69) | 0.021 (-0.44, 0.47) | 0.054 (-0.41, 0.5) | 0.15 (-0.33, 0.56) | -0.13 (-0.55, 0.34) | -0.14 (-0.56, 0.34) |  |
| Free homocysteine | 0.33 (-0.15, 0.68) | 0.13 (-0.34, 0.55) | 0.33 (-0.15, 0.68) | 0.38 (-0.087, 0.71) | 0.36 (-0.11, 0.7) | 0.13 (-0.34, 0.55) | 0.18 (-0.3, 0.59) | 0.26 (-0.22, 0.64) | -0.018 (-0.47, 0.44) | 0.32 (-0.16, 0.68) | 0.44 (-0.017, 0.75) | 0.17 (-0.3, 0.58) | 0.18 (-0.3, 0.59) | 0.25 (-0.23, 0.63) | -0.007 (-0.46, 0.45) | -0.15 (-0.57, 0.32) |  |
| Free reduced homocysteine | -0.081 (-0.52, 0.39) | -0.14 (-0.56, 0.34) | 0.25 (-0.23, 0.63) | 0.054 (-0.41, 0.5) | 0.0053 (-0.45, 0.46) | -0.14 (-0.56, 0.34) | 0.014 (-0.44, 0.47) | -0.067 (-0.51, 0.4) | 0.14 (-0.34, 0.56) | 0.1 (-0.37, 0.53) | 0.16 (-0.31, 0.57) | 0.056 (-0.41, 0.5) | 0.22 (-0.26, 0.61) | -0.014 (-0.47, 0.44) | -0.23 (-0.62, 0.25) | -0.12 (-0.55, 0.35) |  |
| Homocystine | 0.32 (-0.16, 0.67) | 0.24 (-0.24, 0.63) | 0.43 (-0.032, 0.74) | 0.45 (-0.002, 0.75) | 0.34 (-0.13, 0.69) | 0.096 (-0.37, 0.53) | 0.28 (-0.2, 0.65) | 0.23 (-0.25, 0.62) | -0.096 (-0.53, 0.37) | 0.36 (-0.11, 0.7) | 0.51 (0.076, 0.78) | 0.19 (-0.28, 0.6) | 0.081 (-0.39, 0.52) | 0.18 (-0.29, 0.59) | -0.12 (-0.54, 0.36) | -0.1 (-0.53, 0.37) |  |
| Protein-bound homocysteine | 0.12 (-0.35, 0.55) | 0.088 (-0.38, 0.52) | 0.25 (-0.23, 0.63) | 0.37 (-0.1, 0.7) | 0.37 (-0.11, 0.7) | -0.037 (-0.48, 0.42) | 0.35 (-0.12, 0.69) | 0.24 (-0.24, 0.63) | 0.053 (-0.41, 0.5) | 0.22 (-0.26, 0.61) | 0.24 (-0.24, 0.63) | -0.058 (-0.5, 0.41) | 0.012 (-0.44, 0.46) | 0.1 (-0.37, 0.53) | -0.12 (-0.54, 0.36) | -0.16 (-0.57, 0.32) |  |
| Cystathionine | -0.079 (-0.51, 0.39) | -0.068 (-0.51, 0.4) | -0.45 (-0.75, 0.011) | -0.41 (-0.73, 0.049) | 0.058 (-0.41, 0.5) | 0.075 (-0.39, 0.51) | 0.016 (-0.44, 0.47) | -0.13 (-0.55, 0.35) | -0.0053 (-0.46, 0.45) | -0.22 (-0.61, 0.26) | -0.33 (-0.68, 0.14) | 0.054 (-0.41, 0.5) | -0.3 (-0.66, 0.18) | 0.033 (-0.43, 0.48) | 0.11 (-0.36, 0.54) | 0.15 (-0.33, 0.57) |  |
| Total cysteine | 0.35 (-0.13, 0.69) | 0.18 (-0.3, 0.59) | 0.17 (-0.31, 0.58) | 0.18 (-0.29, 0.59) | 0.64 (0.26, 0.85) | 0.41 (-0.056, 0.73) | 0.15 (-0.32, 0.57) | 0.57 (0.16, 0.82) | 0.31 (-0.16, 0.67) | 0.086 (-0.38, 0.52) | -0.19 (-0.59, 0.29) | 0.12 (-0.36, 0.54) | 0.16 (-0.31, 0.58) | 0.36 (-0.12, 0.7) | 0.51 (0.073, 0.78) | -0.27 (-0.65, 0.21) |  |
| Free cysteine | -0.089 (-0.52, 0.38) | -0.29 (-0.66, 0.19) | -0.25 (-0.63, 0.23) | -0.33 (-0.68, 0.14) | 0.15 (-0.33, 0.57) | 0.04 (-0.42, 0.49) | -0.27 (-0.65, 0.21) | 0.028 (-0.43, 0.48) | 0.41 (-0.054, 0.73) | -0.35 (-0.7, 0.12) | -0.032 (-0.48, 0.43) | 0.21 (-0.27, 0.61) | 0.17 (-0.31, 0.58) | -0.0088 (-0.46, 0.45) | 0.3 (-0.18, 0.66) | -0.18 (-0.59, 0.3) |  |
| Free reduced cysteine | -0.39 (-0.72, 0.072) | -0.4 (-0.72, 0.062) | -0.026 (-0.47, 0.43) | -0.27 (-0.64, 0.21) | -0.36 (-0.7, 0.11) | -0.35 (-0.69, 0.12) | -0.43 (-0.74, 0.03) | -0.19 (-0.59, 0.29) | 0.38 (-0.091, 0.71) | -0.39 (-0.71, 0.083) | 0.15 (-0.33, 0.56) | 0.1 (-0.37, 0.53) | 0.61 (0.21, 0.83) | -0.36 (-0.7, 0.11) | -0.26 (-0.64, 0.22) | -0.072 (-0.51, 0.4) |  |
| Cystine | -0.063 (-0.5, 0.4) | 0.12 (-0.36, 0.54) | 0.39 (-0.079, 0.72) | 0.23 (-0.25, 0.62) | 0.23 (-0.25, 0.62) | 0.007 (-0.45, 0.46) | -0.091 (-0.52, 0.38) | 0.45 (0.00022, 0.75) | 0.32 (-0.15, 0.68) | 0.04 (-0.42, 0.49) | 0.056 (-0.41, 0.5) | 0.31 (-0.17, 0.67) | 0.42 (-0.039, 0.74) | -0.12 (-0.55, 0.35) | 0.21 (-0.27, 0.6) | -0.18 (-0.59, 0.3) |  |
| Protein-bound cysteine | 0.5 (0.064, 0.78) | 0.33 (-0.15, 0.68) | 0.33 (-0.14, 0.68) | 0.39 (-0.083, 0.71) | 0.57 (0.16, 0.82) | 0.48 (0.032, 0.77) | 0.26 (-0.22, 0.64) | 0.59 (0.18, 0.82) | 0.12 (-0.35, 0.54) | 0.29 (-0.19, 0.66) | -0.26 (-0.64, 0.22) | -0.025 (-0.47, 0.43) | 0.13 (-0.34, 0.55) | 0.44 (-0.017, 0.75) | 0.44 (-0.019, 0.74) | -0.13 (-0.55, 0.34) |  |
| Total GSH | 0.26 (-0.22, 0.64) | 0.47 (0.025, 0.76) | 0.093 (-0.38, 0.53) | 0.34 (-0.14, 0.69) | 0.24 (-0.24, 0.63) | 0.31 (-0.17, 0.67) | 0.31 (-0.17, 0.67) | 0.17 (-0.31, 0.58) | -0.23 (-0.62, 0.25) | 0.091 (-0.38, 0.52) | -0.17 (-0.58, 0.31) | 0.11 (-0.36, 0.54) | 0.044 (-0.42, 0.49) | 0.026 (-0.43, 0.47) | 0.17 (-0.31, 0.58) | 0.14 (-0.34, 0.56) |  |
| Free GSH | 0.3 (-0.18, 0.66) | 0.29 (-0.19, 0.66) | 0.033 (-0.43, 0.48) | 0.11 (-0.36, 0.54) | 0.07 (-0.4, 0.51) | 0.32 (-0.15, 0.68) | 0.14 (-0.34, 0.56) | -0.0018 (-0.46, 0.45) | -0.27 (-0.65, 0.21) | 0.1 (-0.37, 0.53) | 0.14 (-0.34, 0.56) | 0.23 (-0.25, 0.62) | -0.11 (-0.54, 0.36) | 0.072 (-0.4, 0.51) | 0.18 (-0.3, 0.59) | 0.15 (-0.33, 0.56) |  |
| GSH | 0.34 (-0.14, 0.69) | 0.33 (-0.15, 0.68) | -0.014 (-0.47, 0.44) | 0.21 (-0.27, 0.6) | 0.051 (-0.41, 0.49) | 0.28 (-0.2, 0.65) | 0.12 (-0.36, 0.54) | 0.014 (-0.44, 0.47) | -0.28 (-0.65, 0.2) | 0 (-0.45, 0.45) | 0.058 (-0.41, 0.5) | 0.19 (-0.29, 0.59) | -0.12 (-0.54, 0.35) | 0.042 (-0.42, 0.49) | 0.21 (-0.27, 0.61) | 0.29 (-0.18, 0.66) |  |
| GSSG | 0.49 (0.045, 0.77) | 0.44 (-0.017, 0.75) | 0.18 (-0.3, 0.59) | 0.06 (-0.41, 0.5) | 0.24 (-0.24, 0.62) | 0.45 (-0.0064, 0.75) | 0.39 (-0.079, 0.72) | -0.03 (-0.48, 0.43) | -0.18 (-0.59, 0.3) | 0.32 (-0.16, 0.68) | -0.0018 (-0.46, 0.45) | 0.36 (-0.11, 0.7) | -0.5 (-0.78, -0.064) | 0.34 (-0.14, 0.69) | 0.41 (-0.054, 0.73) | 0.39 (-0.081, 0.72) |  |
| Protein-bound GSH | 0.2 (-0.28, 0.6) | 0.35 (-0.12, 0.69) | 0.14 (-0.33, 0.56) | 0.4 (-0.068, 0.72) | 0.32 (-0.16, 0.67) | 0.22 (-0.26, 0.62) | 0.22 (-0.26, 0.61) | 0.34 (-0.13, 0.69) | 0.028 (-0.43, 0.48) | 0.098 (-0.37, 0.53) | -0.31 (-0.67, 0.17) | 0.021 (-0.44, 0.47) | 0.093 (-0.38, 0.53) | 0.091 (-0.38, 0.52) | 0.23 (-0.25, 0.62) | 0.025 (-0.43, 0.47) |  |
| Taurine | 0.32 (-0.16, 0.67) | 0.3 (-0.18, 0.66) | 0.35 (-0.12, 0.69) | 0.46 (0.014, 0.76) | 0.22 (-0.26, 0.61) | 0.24 (-0.25, 0.62) | 0.2 (-0.28, 0.6) | 0.56 (0.15, 0.81) | 0.11 (-0.36, 0.54) | 0.21 (-0.27, 0.6) | -0.042 (-0.49, 0.42) | -0.021 (-0.47, 0.44) | 0.35 (-0.12, 0.7) | 0.2 (-0.28, 0.6) | 0.31 (-0.17, 0.67) | 0.15 (-0.32, 0.57) |  |
